# In-vitro human myogenesis model reveals novel mRNA alternative splicing isoforms

**DOI:** 10.1038/s41598-025-16523-2

**Published:** 2025-10-01

**Authors:** Stefano Donega, Nirad Banskota, Jen-Hao Yang, Martina Rossi, Yulan Piao, Dimitrios Tsitsipatis, Jinshui Fan, Supriyo De, Charlotte A. Peterson, Mary M. McDermott, Myriam Gorospe, Luigi Ferrucci

**Affiliations:** 1 Translational Gerontology Branch, National Institute on Aging, NIH, Baltimore, MD USA; 2 Laboratory of Genetics and Genomics, National Institute on Aging, NIH, Baltimore, MD USA; 3 Center for Muscle Biology, College of Health Sciences, University of Kentucky, Lexington, KY USA; 4 Department of Medicine, Northwestern University Feinberg School of Medicine, Chicago, IL USA; 5 National Institute on Aging Intramural Research Program, Biomedical Research Center, National Institutes of Health, 251 Bayview Boulevard, Suite 100, Baltimore, MD 21224 USA

**Keywords:** In-vitro muscle model, Myogenesis, mRNA, Splicing, AI-neural network, RNA splicing, Transcriptomics

## Abstract

Myogenesis, the process of muscle formation and regeneration, involves substantial alterations in gene expression. While alternative splicing plays a crucial role in generating proteomic diversity during development and disease, its specific contributions to human muscle differentiation have not been systematically explored. Here, we examined altered mRNA splicing during myogenesis in two human myoblast cell lines using a hybrid transcriptomic approach that combines short-read (Illumina) and long-read (Nanopore) RNA-seq analyses. We identified 13,853 new significant splicing isoforms (60,582 total), with RNAs increasing and decreasing in abundance between days 0 and 3 (3,771 and 3,649, respectively), and between days 3 and 5 (1,302 and 1,109, respectively). We identified 1,937 significant differential transcript usage events (DTUs), implicating pathways relevant for muscle regulation. These findings were validated using RT-qPCR analysis and across mouse and human models, including clinical samples from peripheral artery disease patients. Artificial Intelligence algorithms predicted 595 myogenesis-associated, high-confidence, novel protein-coding splicing isoforms. This study uncovers splicing-regulated mechanisms in muscle development and pathologies, establishing an integrative framework for studying mRNA processing, essential for future muscle biology intervention studies.

**Supplementary Information:**

The online version contains supplementary material available at 10.1038/s41598-025-16523-2.

## Background

The regulation of gene expression is a complex and multifaceted process governed by tightly coordinated regulatory networks^1^. Central to this process is alternative splicing, a highly orchestrated process involving cis-acting RNA sequences and over 150 trans-acting factors that give rise to different mRNAs encoding alternative forms of proteins from the same gene^2^. Myogenesis, the process whereby skeletal muscle forms during embryonic development and regenerates throughout life, is coordinated by closely regulated gene expression programs^3,4^. During myogenesis, myoblasts fuse to form multinucleated myofibers^5,6^, and this process is critical for the formation of embryonic and postnatal muscle tissue. Myogenic cell differentiation is affected by extrinsic and intrinsic factors, including morphogen gradients and signal transduction pathways^7–13^.

Alternative splicing plays an important role in myogenesis by expanding the diversity of proteins generated from a limited number of genes. This process is orchestrated by RNA-binding proteins (RBPs) regulated through temporal expression patterns and posttranslational modifications^14^, which control the splicing of pre-mRNAs encoding proteins critical for muscle development and function^15,16^. Splicing factors such as polypyrimidine tract-binding protein 1 (*PTBP1*) have been linked to splicing regulation during tissue regeneration and in muscle pathologies^17^. Recent studies have uncovered the complexity of myogenesis, particularly adult myogenesis, in aging, as it is characterized by weakened myogenic differentiation and increased pro-apoptotic states^18,19^. There is some evidence that the age-associated myogenic impairment combined with decreased growth factor expression and diminished muscle stem cell regenerative capacity leads to sarcopenia^20–23^. Targeted metabolic reprogramming has been shown to counter mitochondrial dysfunction, which may be implicated in impaired myogenesis and age-related skeletal muscle loss^24,25^.

Transcriptomic analyses have revealed distinct mRNA profiles in satellite cells that regulate their quiescence, activation, and differentiation. These tightly controlled transcriptomes point to the precise regulation of gene expression programs driving adult muscle development and regeneration. However, there is limited information on the temporal appearance of single splicing isoform variants during these processes. Studies that compared whole muscle tissue from young and old individuals found that RNA processing and splicing are among the most differentially regulated biological processes during aging. Investigating these processes across life stages, and in particular the role of protein isoforms in the maintenance and repair of muscle integrity and function with aging, is crucial for a deeper understanding of muscle regeneration and could identify possible new targets for age-related sarcopenia prevention.

Our analysis utilizes a multi-leveled computational approach that incorporates information from short- and long-read sequencing (hybrid platform). The use of an unbiased Likelihood Ratio-based clustering method allowed the characterization of complex isoform changes during myogenesis, while it also allowed us to identify specific transcript isoforms contributing to the global expressed transcriptome. By combining differential transcript usage (DTU) analysis—which detects changes in mRNA splicing isoform proportions across conditions or time points—with single-isoform variant expression analysis and AI-based tools like ESMFold and AlphaPulldown, we identify splicing isoforms and mRNAs that likely encode key proteins involved in myogenesis.

## Methods

### Study design, cell culture and RNA collection

Human Immortalized myoblast cell lines AB1167 and AB678 were provided by Dr. Gorospe Lab (NIA, IRP) and developed as previously reported^26,27^. Cells were cultured in Ham’s F10 media with 20% Fetal Bovine Serum (FBS) and Promocell Skeletal Muscle Cell Growth Medium^28,29^. AB678 and AB1167 myoblasts were cultured in proliferative conditions (day 0) and then induced to differentiate by culturing them to high density and replacing the growth medium with differentiation medium, Dulbecco’s modified Eagle medium (DMEM) containing 2% horse serum. RNA was collected every 24 h over the 5-day differentiation period.

### Reverse transcription and quantitative polymerase chain reaction (RT-qPCR) analysis

For RT-qPCR analysis, RNA from AB1167 and AB678 cells (500 ng each) was reverse-transcribed to cDNA using Maxima Reverse Transcriptase (Thermo Fisher Scientific) and random hexamers. To distinguish sense and antisense transcripts, the first-strand synthesis was performed with specific primers. RT-qPCR analysis was conducted with SYBR green master mix (Kapa Biosystems) on a QuantStudio 5 Real-Time PCR System (Thermo Fisher Scientific) after measuring *GAPDH* mRNA levels for normalization using the 2^−ΔΔCt^ method. Primer sequences are listed (Supplementary Material 1).

### Library Preparation and hybrid RNA sequencing

Total RNA collection included a digestion step using DNase I, extracted using Direct-zol™ RNA MiniPrep kit (Zymo Research). RNA quality and quantity were checked using Agilent RNA Screen Tape on the Agilent Tapestation.

For short-read (Illumina) sequencing, libraries were prepared from 150 ng RNA using an Illumina TruSeq Stranded mRNA Library prep kit following the manufacturer’s protocol (Illumina, Cat#20020594). After cDNA generation, the cDNAs were subjected to 3’-end adenylation, adapter ligation, and purification with AMPure beads (Beckman, Cat#A63881). The products were size-selected with SPRIselect beads (Beckman, Cat#B23318), and the selected cDNAs enriched by PCR and purified again with SPRIselect beads to generate final libraries. The quality and quantity of the sequencing libraries were assessed using Agilent DNA 1000 Screen Tape on the Agilent Tapestation. Paired-end sequencing was performed for ~ 110 cycles using an Illumina NovaSeq 6000 instrument.

For long-read (Nanopore) sequencing, 75 ng of RNA were used to prepare the sequencing library using a cDNA-PCR Sequencing Kit (SQK-PCS109) following the manufacturer’s protocol (Oxford Nanopore). The quality and quantity of sequencing libraries were assessed using Agilent High Sensitivity DNA kit (Agilent, Cat#5067 − 4626) on the Agilent Bioanalyzer. Sequencing was performed using a GridION instrument at a depth of ~ 20 million reads per sample.

RNA Sequencing (both short- and long- reads) was performed at the National Institute on Aging. Nanopore was base-called using Guppy v. 6.0.1. Short-read data were processed by checking quality (FastQC v. 0.11.8, https://www.bioinformatics.babraham.ac.uk/projects/fastqc/), trimming (BBDuk, https://jgi.doe.gov/Data-and-tools/software-tools/bbtools/), and mapping to the human genome 38 and annotation version 104 using STAR (v. 2.7.10a). Minimap version 2.17 was used for mapping long-read Data. For hybrid assembly and quantification of isoforms, Stringtie2 (v. 2.1) with the –mix parameter was used to combine long-read and short-read data. SQANTI3 (v. 4.3) was used to annotate known and novel transcripts.

### Differential aggregated RNA and differential isoform expression analysis

DESeq2 (v. 1.36.0) was used for performing differential aggregated RNA (DGE) or differential isoform expression (DIE)^30^. Hierarchical clustering was used to group both RNAs and isoforms showing similar patterns. Statistical significance was set at adjusted p-value less than 0.05.

### Differential isoform usage analysis

To perform differential transcript/isoform usage analysis (DTU or DIU), DRIMSeq^31^ was used to filter isoforms using the following parameters: min_samples_feature_expr = 18, min_samples_feature_prop-adj = 18, min_samples_gene_expr = 18, min_feature_expr = 5, min_feature_prop-adj = 0.05, and min_gene_expr = 10. DEXseq (v. 1.42.0)^32^ was then utilized for differential isoform usage analysis. Briefly, Size Factors and dispersions were estimated by using estimateSizeFactors() and estimateDispersions() functions respectively. To test for differential isoform usage, we utilized the testforDEU() function and adjusted for cell line. Finally, StageR^33^, which uses a two-stage testing procedure (transcripts mapping to the same gene and specific isoforms) for controlling false discovery was utilized for significance testing at an alpha of 0.05.

### Gene set enrichment analysis

ClusterProfiler 4.4.4 was utilized for gene set enrichment analysis (GSEA) and Over Representation Analysis (ORA)^34^. In contrast to GSEA, which takes the entire Dataset as input, ORA only utilizes those subsets of genes identified through the analysis of differentially abundant transcripts which were best fitting given our isoforms input. ORA of isoform usage was performed using a subset of the output of the model using a threshold of padj < 0.05 with detected RNAs used for background. We considered as significant only those ontologies with p-adjusted (p-adj) < 0.05. The package ggtranscript v. 0.99.9^35^ was used for visualizing the structure of novel RNAs and isoforms.

### Mitochondrial scores

To calculate mitochondrial scores for each complex, we utilized the MitoCarta database. Briefly, we first included only those genes associated with their respective complex. Next, the count matrix was normalized using Trimmed Mean of M-values (TMM), Counts Per Million (CPM), and logarithmic transformations via the edgeR package. Effects due to the cell line was removed using the "removeBatchEffect()" function from the limma packages. Each gene was subsequently z-score normalized to ensure that each gene had comparable expression scale. Finally, mitochondrial scores were calculated by averaging gene expression values for each sample. Statistical significance and effect size ( or the Beta) were calculated using the lm() function. For the glycolysis score, we manually curated the gene set, and applied the same approach described above. To calculate myogenesis score, we first subset genes within each specific cluster, and scores were calculated by averaging normalized expression within relevant datasets such as PAD or mice.

### Structure prediction using ESMFold and alphapulldown

SQANTI3 utilizes GeneMarkS-T to identify isoforms that may have regions that are protein-coding^36^. To confidently identify novel coding isoforms, we ran ESMFold^37^ to predict the structure of all predicted novel protein-coding isoforms identified by GeneMarkS-T. ESMFold uses a large language model (LLM) for predicting the structure of a protein, and is faster than AlphaFold, allowing us to predict the structure of more than 17,000 novel isoforms. The pLDDT score is a confidence score ranging from 0 to 100, where 0 is no confidence and 100 is the highest confidence. By using ESMFold to classify only those isoforms that could be reliably predicted, we enhanced the transcript biotype classification by categorizing novel isoforms into three groups: known coding (for novel isoforms encoding a known protein), high-confidence coding, or low-confidence coding.

To confidently identify novel protein-coding isoforms, we first utilized Sqanti3 to predict amino acid sequences. Next, to detect protein-protein interactions, we used the top 50 protein-coding genes (here we focused on LRT, likelihood ratio test), with the lowest adjusted p-value to run AlphaPulldown^38^, which uses Colabfold^39^ internally. For a single protein, 50 interactions (including self-interaction for identifying homodimers) were computed. We used iPTM (interface predicted template modeling) score - a confidence metric in protein-protein interaction prediction - threshold greater than 0.7 to identify highly confident interactions. These interactions were visualized as a network graph and as a heatmap.

Figures were created using Graphpad Prism (version 10.1.1), ggplot2 package in R, Microsoft Office 365 (version 16.92) and BioRender (www.biorender.com). The software version of all Rstudio software used in this study are provided (Supplementary Material 2). A flow diagram of bioinformatic tools is provided as visual summary (Supplementary Material 3).

## Results

### Hybrid transcriptomic analysis across human myogenesis

**Fig. 1:**
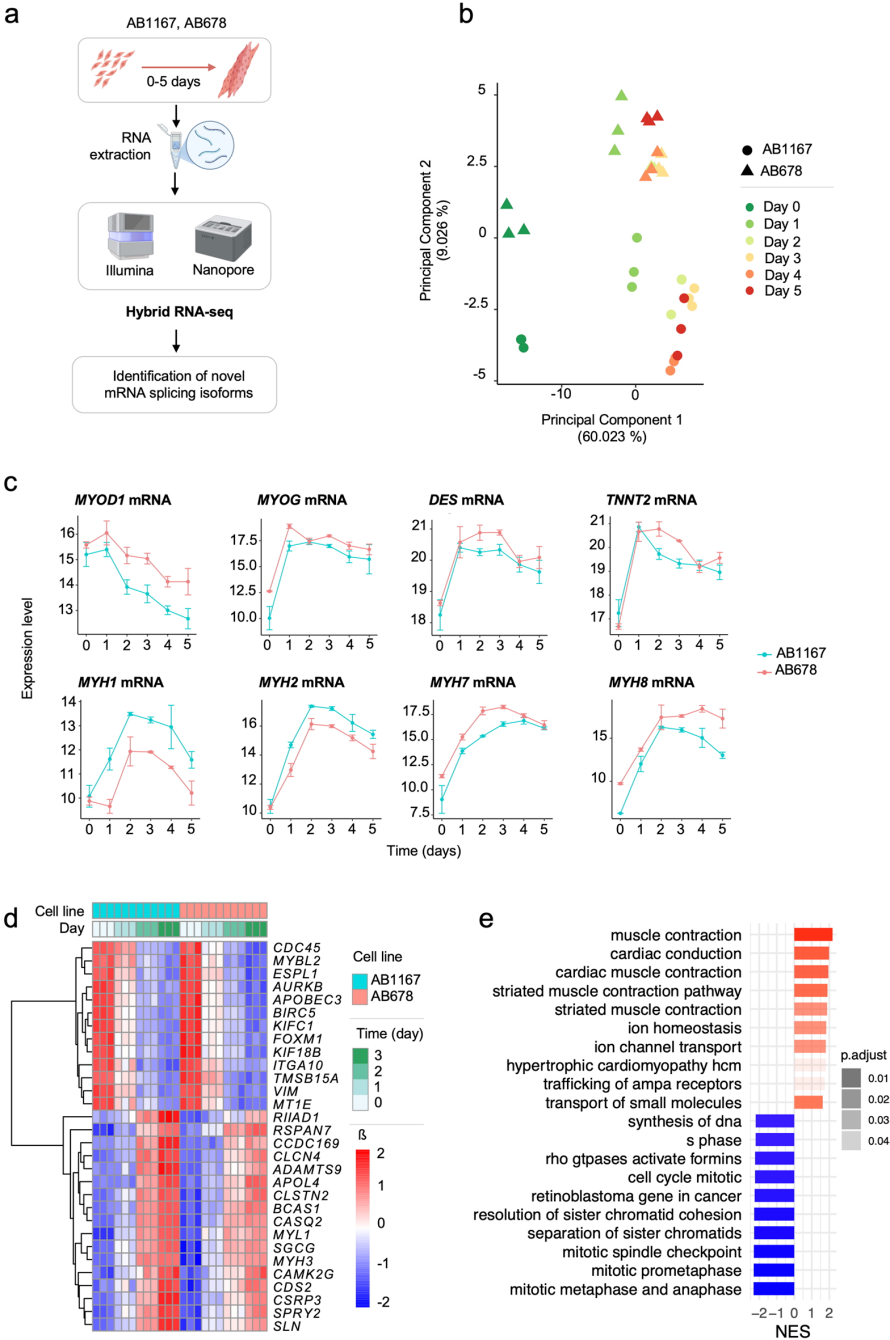
Transcriptomic changes in muscle differentiation. **(a)** Representative experimental design. **(b)** Principal Component Analysis (top 100 mRNAs species): colors indicate progression across stages of muscle differentiation; shapes denote different cell lines. **(c)** Myogenesis markers mRNA expression levels using normalized expression Data from hybrid RNA-seq. Each color represents one of the two myoblast lines: AB1167 (cyan) and AB678 (coral). **(d)** Linear Regression model (0–3 days): Heatmap representing top 15 mRNAs more and less abundant in each cell line. **(e)** Linear Regression model (0–3 days): Gene Set Enrichment Analysis (GSEA) representing top 10 biological pathways overrepresented and underrepresented.

Transcriptomic analysis was conducted in two human myoblast cell lines, AB678 and AB1167, as they differentiated from myoblasts to myotubes over a 5-day period in culture. Analysis was performed using short-read (Illumina) and long-read (Oxford Nanopore) sequencing technologies (Fig. 1a); short-reads provided high accuracy for splice site validation, while long-reads captured full-length transcripts spanning multiple exons. This integration leveraged the strengths of both technologies, improving splice junction detection and enabling the reconstruction of complete transcripts. The hybrid computational approach outperformed single-platform approaches in detecting mRNA isoforms, identifying 60,582 splicing isoforms, including 13,853 new ones. Of these new variants, 7,826 were characterized by the presence of previously annotated splice sites (in catalog), while 6,027 were entirely new variants never annotated before (not in catalog), significantly expanding previous mRNA catalogs (Supplementary Material 4). This approach maximized the detection of “full-splice match” transcripts and enhanced the identification of antisense and fusion RNAs. Given these advantages in both quantitative and qualitative detection, Data analyses were carried out by integrating these platforms through a hybrid approach in all subsequent analyses.

Principal component analysis (PCA) was employed to assess transcriptomic dynamics during human myogenic differentiation and to evaluate overall RNA expression differences between the two cell lines (Fig. 1b). The most pronounced transcriptomic shift occurred between days 0 and 1. After this initial shift, the changes became more gradual from days 2 to 5. This pattern is clearly visible along principal component 1, where the data points for days 2 to 5 are clustered closely together, while principal component 2 primarily reflected the inherent variance between different cell lines.

We analyzed the time-dependent expression of eight canonical mRNA markers of myogenesis in both muscle cell lines, every 24 hours during the 5-day experimental design (Fig. 1c). In both cell lines, we specifically assessed the expression levels of *MYOD1* mRNA (encoding a key regulator of myoblast proliferation), *MYOG* mRNA (encoding a transcription factor essential for myoblast differentiation and myotube formation), *DES* mRNA (encoding a muscle-specific type-III intermediate filament important for sarcomere structure), and *TNNT2* mRNA (encoding a protein implicated in Ca^2+^-dependent regulation of striated muscle contraction). Additionally, we assessed the expression of myosin heavy chain isoforms encoded by *MYH1* and *MYH2* mRNAs (for fast-twitch), *MYH7* mRNA (for slow-twitch), and *MYH8* mRNA (a perinatal isoform). These markers confirmed the expected changes in the production of key transcriptional regulators and structural proteins involved in diverse phases of myogenic differentiation and muscle fiber specialization. We detected similar expression trends between the two cell lines and, therefore, in subsequent analysis we used statistical models that accounted for cell line as a covariate instead of analyzing each cell line separately. To strengthen the validity of our findings, we corroborated the expression patterns of our identified markers by analyzing public data. Accordingly, we extracted data from a proteomic study of myogenesis conducted in mouse C2C12 myoblast cell lines^40^. We confirmed the overexpression trend for four matched proteins -namely Tnnt2, Des, Myh7, and Myh8 - thereby reinforcing the consistency of our results (Supplementary Material 5a).

Using the list of mitochondrial mRNA protein-coding genes from the MitoCarta 3.0 Dataset, we calculated composite scores for mitochondrial oxidative phosphorylation (OXPHOS) and glycolysis by averaging the expression levels of relevant nuclear and mitochondria-encoded mRNAs at each time point, covering days 0 to 5 (Supplementary Material 5b). The temporal trends and significance of these mitochondrial scores were analyzed across the entire differentiation period (days 0 to 5), and in subintervals (days 0 to 3, and 3 to 5). Focusing on days 0 to 3, we found increased levels of mRNAs encoding proteins involved in electron transport chain complexes I (*p* = 0.048, β = 0.13), II (*p* = 0.046, β = 0.17), and IV (*p* = 0.004, β = 0.18). Although the changes were not statistically significant from days 0 to 3 (*p* = 0.37), those mRNAs encoding mitochondrial complex V proteins tended to increase from days 0 to 1. In contrast, changes of mRNAs encoding protein components of complexes III did not exhibit statistically significant differences. The glycolysis score did not show significant changes in either phase. Of note, the abundance of mRNAs encoding glycolytic enzymes declined in the early phase (*p* = 0.11, β=-0.07) and increased during the late phase when myotube formation commenced (*p* = 0.15, β = 0.21). These contrasting trends suggest a metabolic shift as myogenesis progresses from myoblast proliferation to myotube formation.

To capture two distinct “early and late” phases of differentiation, we used two linear regression models: one covered the “canonical” muscle differentiation phase (days 0 to 3) and the second the post-differentiation phase (days 3 to 5). Differential expression analysis of the early myogenesis phase (days 0 to 3) identified 3,771 upregulated and 3,649 downregulated mRNAs (p-adjusted < 0.05; Fig. 1d, and Supplementary Material 6, sheet 1). GSEA in days 0 to 3 identified 10 pathways associated with the proteins encoded by the elevated mRNAs, primarily associated with muscle contraction and metabolic processes, while the 113 pathways less represented included mRNAs encoding proteins involved in cell cycle phase transitions and DNA synthesis (Fig. 1e and Supplementary Material 6, sheet 2).

**Fig. 2:**
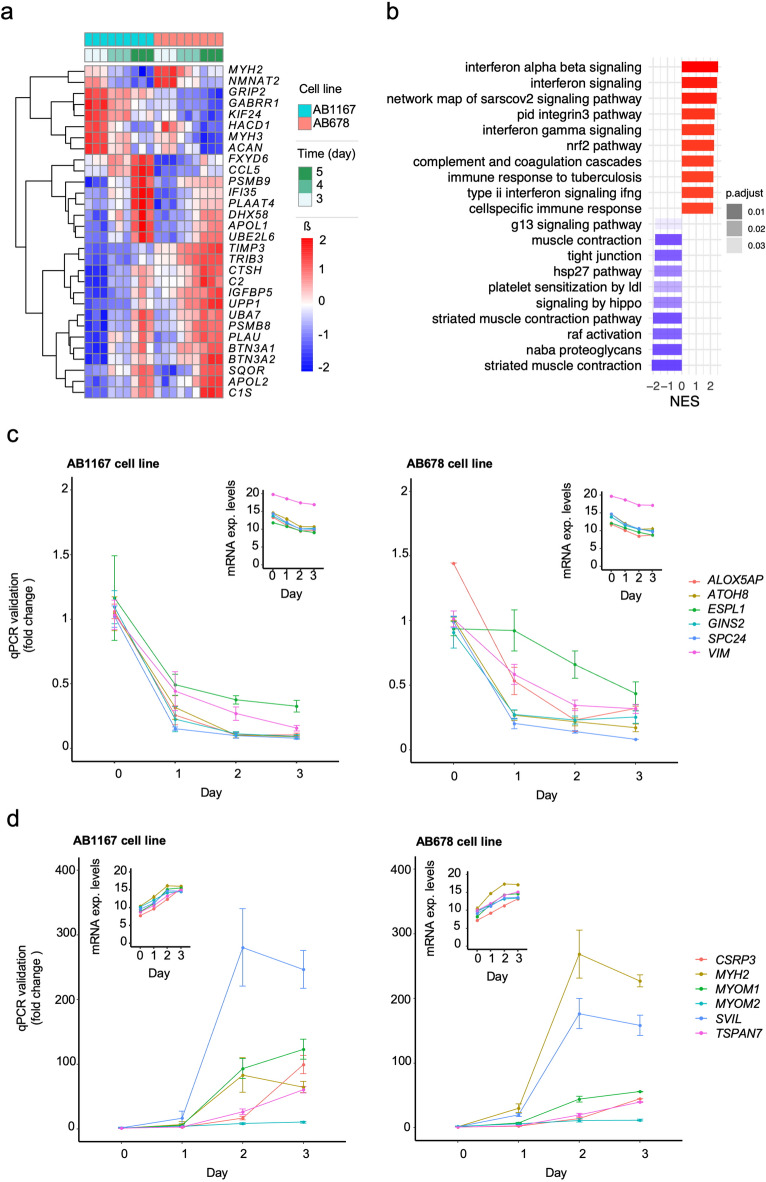
Late-myogenesis mRNA changes and validation of Linear regression models. **(a)** Linear Regression model (3–5 days): Heatmap representing top 15 mRNAs more and less abundant in each cell line. **(b)** Linear Regression model (3–5 days): Gene Set Enrichment Analysis (GSEA) representing top 10 biological pathways overrepresented and underrepresented. **(c)** Validation of lower expressed mRNAs in AB1167 (left) and AB678 (right) cell lines using RT-qPCR. One representative mRNA per expression cluster, normalized to *GAPDH* mRNA, is shown for each cell line, with genes selected based on p-adj < 0.001 (*ALOX5AP*,* ATOH8*,* ESPL1*,* GINS2*,* SPC24*,* VIM*). **(d)** Validation of higher expressed mRNAs in AB1167 (left) and AB678 (right) cell lines using RT-qPCR. One representative mRNA per expression cluster, normalized to *GAPDH* mRNA, is shown for each cell line; mRNAs were selected based on p-adj < 0.001 (*CSRP3*,* MYH2*,* MYOM1*,* MYOM2*,* SVIL*,* TSPAN7* mRNAs).

The late phase of differentiation (days 3 to 5) showed milder shifts, with 1,302 increased and 1,109 decreased mRNAs (Fig. 2a and Supplementary Material 6, sheet 3). GSEA for those mRNAs more abundant in late phase represented 84 pathways predominantly linked to interferon signaling and inflammatory responses, possibly reflecting cellular stress due to culture conditions (Fig. 2b and Supplementary Material 6, sheet 4). The 15 less represented pathways in this phase did not show clear biological relevance and will require further investigation.

We then validated the expression changes detected by hybrid RNA sequencing through reverse transcription and quantitative polymerase chain reaction (RT-qPCR) analysis of 12 representative mRNAs (p-adj < 0.001) in both cell lines during days 0 to 3, with expression levels normalized to *GAPDH* mRNA levels. We validated the following downregulated mRNAs (Fig. 2c): *ALOX5AP* mRNA (encoding a leukotriene biosynthesis protein, p-adj = 2e^− 19^, β=-0.053), *ATOH8* mRNA (encoding a protein important for transcriptional regulation during development, p-adj = 2e^− 26^, β=-0.059), *ESPL1* mRNA (encoding a protein implicated in chromosome segregation, p-adj = 1e^− 88^, β=-0.048), *GINS2* mRNA (encoding a DNA replication protein, p-adj = 6e^− 30^, β=-0.054), *SPC24* mRNA (encoding a protein with key roles in kinetochore-microtubule attachment, p-adj = 4e^− 38^, β=-0.067), and *VIM* mRNA (encoding a cytoskeletal organization protein, p-adj = 3e^− 50^, β=-0.038). We also validated the following upregulated mRNAs (Fig. 2d): *CSRP3* mRNA (encoding a protein important for myogenesis and cardiac stress signaling, p-adj = 2e^− 213^, β = 0.116), *MYH2* mRNA (encoding a fast-twitch myosin heavy chain, p-adj = 1e^− 33^, β = 0.095), *MYOM1* and *MYOM2* mRNAs (encoding sarcomere M-band protein components, p-adj = 4e^− 48^, β = 0.108 and p-adj = 1e^− 46^, β = 0.061, respectively), *SVIL* mRNAs (encoding actin-binding and cytoskeletal organization proteins, p-adj = 4e^− 40^, β = 0.08), and *TSPAN7* mRNA (encoding a protein important in development, p-adj = 2e^− 112^, β = 0.088). These results corroborate the presence of distinct molecular mechanisms and regulatory networks operating throughout the myogenic program, as initially indicated by the RNA-seq analysis.

### mRNAs fluctuations, AI-predicted protein-protein interactions, and myogenesis score comparison to mouse myogenesis and human muscle biopsies

**Fig. 3:**
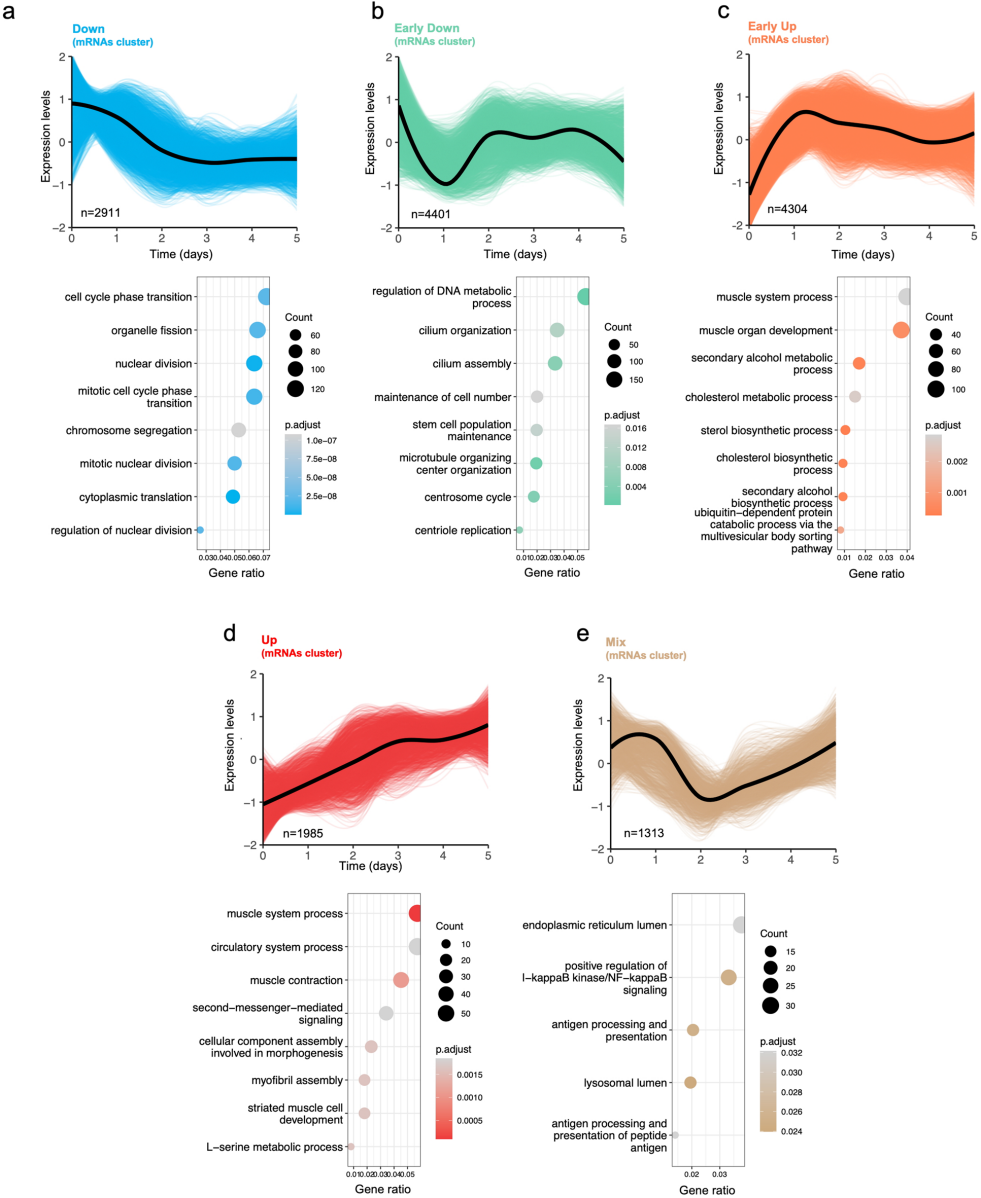
mRNA cluster identification during myogenesis. Identification of five mRNA clusters during full model (0–5 days) myogenesis using a Hierarchical Clustering- Likelihood Ratio Test (HC-LRT) approach). Top panels represent the dynamics of the cluster identified. Bottom panel: Pathway analysis for the clusters identified using Over-Representation Analysis (ORA) representing top 10 pathways associated to myogenesis. **(a)** Down mRNA cluster (2911 mRNAs identified). **(b)** Early Down mRNA cluster (4401 mRNAs identified). **(c)** Eary Up mRNA cluster (4304 mRNAs identified). **(d)** Up mRNA cluster (1985 mRNAs identified). **(e)** Mix mRNA cluster (1313 mRNAs identified).

To analyze non-linear expression profiles over the 5-day myogenesis paradigm, we used a hierarchical clustering-likelihood ratio test (HC-LRT) (Supplementary Material 6, sheet 5). This analysis identified significant differentially expressed mRNAs (p-adj ≤ 0.01) grouped into five distinct temporal clusters: “Down” (n = 2911, declining linearly up to day 3, Fig. 3a), “ Early-Down” (*n* = 4401, dramatically declining the first day, Fig. 3b), “Early-Up” (*n* = 4304, immediately increased, then stable, Fig. 3c), “Up” (*n* = 1985, increasing up to day 3, Fig. 3d), and “Mix” (*n* = 1313, no clear trend, Fig. 3e).

Over-representation analysis (ORA) was used to elucidate the functional significance of these mRNA clusters (Fig. 3a-e lower panels and Supplementary Material 6, sheet 6). Both “Down” and “ Early-Down” clusters were enriched in mRNAs encoding cell cycle and mitotic nuclear division proteins, including several involved in cellular replication (194 and 53 enriched pathways identified, respectively). Notably, the “Early-Up” mRNA cluster was enriched in 103 pathways, mainly involved in alcohol, cholesterol, and sterol biosynthesis metabolic processes. The “Up” mRNA cluster mainly identified muscle-related processes among 74 enriched pathways, with a linear trend up to day 3 of differentiation. The “Mix” mRNA cluster was not uniform and included 5 biological pathways critical for adaptive immune responses, such as antigen processing and presentation, essential for immune system recognition and response to pathogens and abnormal cells.

**Fig. 4:**
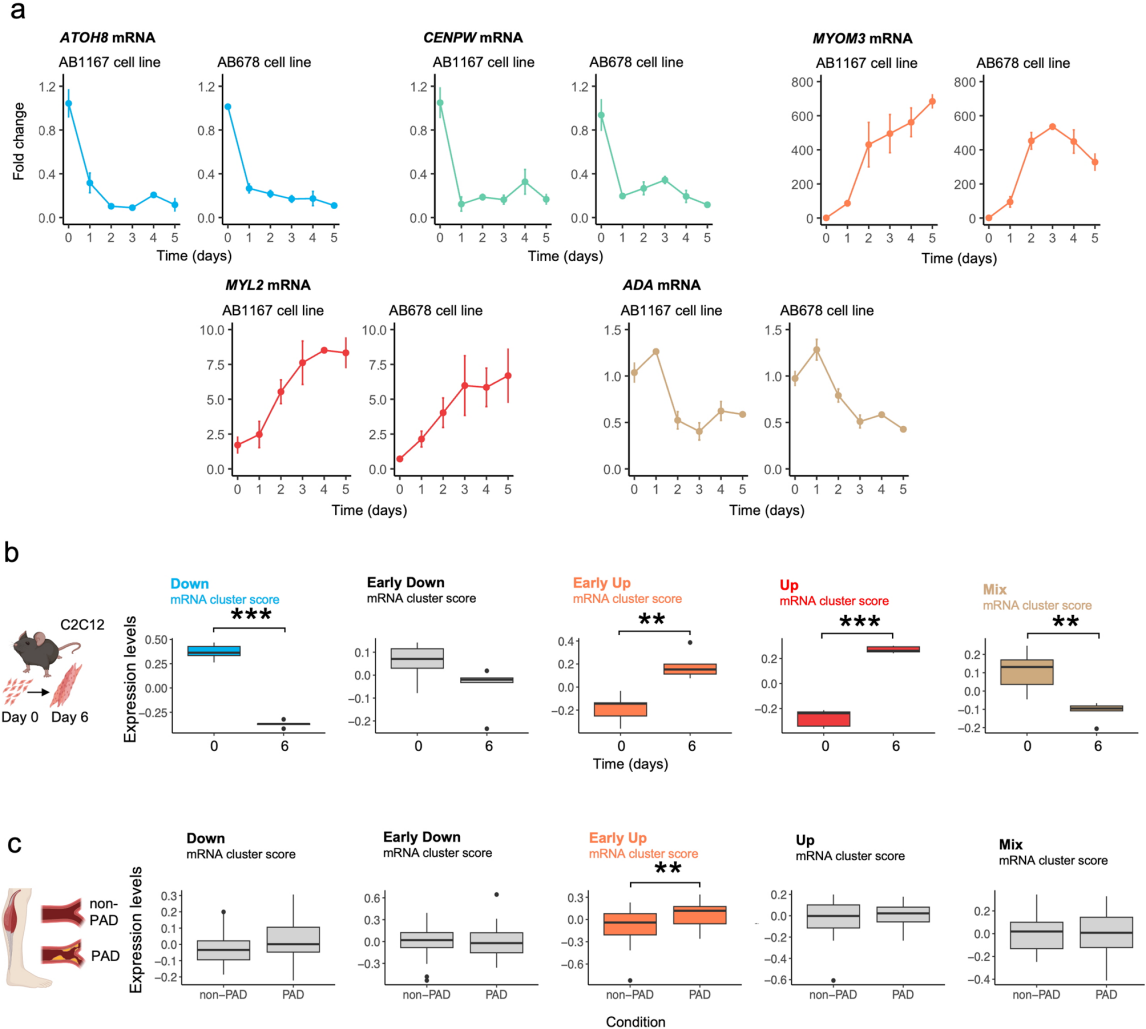
Validation for HC-LRT using RT-qPCR and across independent studies in mice muscle cell and in-vivo in human muscle Peripheral Artery Disease. **(a)** RT-qPCR clusters validation for HC-LRT mRNA clusters in AB1167 and AB678 cells, normalized to *GAPDH* mRNA. For “Down” cluster: *ATOH8* mRNA; for “Early-Down” cluster: *CENPW* mRNA; for “Early-Up” cluster: *MYOM3* mRNA; for “UP” cluster: *MYL2* mRNA; for “Mix” cluster: *ADA* mRNA. **(b)** Validation using transcriptomes from mouse myogenesis (C2C12 cells Dataset) reported earlier^41^. **(c)** Validation using transcriptomes from human peripheral artery disease (PAD Dataset, human muscle biopsies) from another study^42^. For **b)** and **c)**, statistical significance was determined using appropriate tests, with p-adj indicated as follows: p-adj < 0.05 (p-adj < 0.05:*; p-adj < 0.01: **; p-adj < 0.001: ***). Color-matching with mRNA cluster when p-adj < 0.05.

Representative mRNAs from each myo-mRNA clusters were selected for subsequent RT-qPCR validation in both cell lines (Fig. 4a): *ATOH8* mRNA (encoding a protein important for transcription during development, HC-LRT p-adj = 9e^− 197^; DGE p-adj = 2e^− 26^, β=-0.059), *CENPW* mRNA (encoding a protein important for cell division, HC-LRT p-adj = 1e^− 187^; DGE p-adj = 4e^− 9^, β=-0.03), *MYOM3* mRNA (encoding a protein component of the M-band of the sarcomere, HC-LRT p-adj = 0; DGE *p* = 0.1, p-adj = 0.11, *p* = 0.04, β = 0.01), *MYH2* mRNA (encoding a protein for fast-twitch skeletal muscle-type IIx/IIa fibers, HC-LRT p-adj = 1e^− 122^; DGE p-adj = 1e^− 33^, β = 0.09), and *ADA* (encoding a protein for purine metabolism and adenosine homeostasis, HC-LRT p-adj = 3e^− 56^; DGE p-adj = 1e^− 10^, β=-0.03).

Next, we investigated whether the differential transcriptomic patterns identified with myogenesis were related to specific biological functions. To this end, we analyzed AI-generated protein neural network based on amino acid sequences. We used predictions from SQANTI3 to convert mRNA sequences into hypothetical protein amino acid sequences. From these protein sequences, we selected the top 50 showing the largest expression changed with myogenesis and had protein sequences included in the Uniprot and Swissprot Database. We then employed Alphapulldown, which internally uses Colabfold to evaluate protein-protein interactions among these proteins. We tested 1,275 total interactions among these 50 mRNA protein-coding genes and identified 19 myogenesis-associated proteins distributed across 7 distinct fully connected components, 3 of which contained just 1 protein forming homodimers (Supplementary Material 7b derived from the cross-correlation comparison in Supplementary Data 7a). Three clusters, comprising 6 proteins, were linked to muscle formation, development, and contraction. One cluster of 6 proteins was associated with fatty acid metabolism, WNT signaling, and calcium channels. Another cluster containing 5 proteins was related to actin-binding protein degradation, cell division, and sarcomere integrity. Additionally, two single-protein clusters were identified, one involved in metal ion regulation and the other in calcium ATPase activity. Supplementary material 7c represent the change in expression for these items across myogenesis.

To further validate our findings and to compare the transcriptomic patterns to those identified in published articles, we developed a “myogenesis score” based on enriched mRNAs from each of these five identified clusters and tested this score against the transcriptomes reported in three independent studies. In the first study, mouse C2C12 myoblasts were analyzed at days 0 and 6 of differentiation^41^. Except for the “ Early-Down” cluster, which exhibited marginal significance (*p* = 0.09), all other four myo-score clusters showed a significant fit (Fig. 4b). In the second study, we examined transcriptomes from muscle biopsies of patients with peripheral artery disease (PAD)^42^. The “Early-Up” mRNA cluster was the only one significantly different between PAD and controls (*p* = 0.008), supporting the hypothesis that muscle tissue under pathological stress from ischemia undergoes damage and regeneration events (Fig. 4c). Conversely, the downregulated cluster displayed an inverse trend (*p* = 0.13), with mRNAs encoding proteins associated with cell division and replication.

In the third study, we analyzed transcriptomes from human muscle biopsies taken from participants in the ‘healthy aging’ GESTALT cohort^43^. No significant associations were found between our cluster-derived scores and physical fitness (as measured by VO_2_ peak in young and old donor) or chronological age, as analyzed across low and high VO_2_ peak tertiles (Supplementary Material 8).

### Alternatively spliced mRNA during myogenesis

To identify significant switches in mRNA splicing isoforms during differentiation, we performed a Differential Transcript Usage (DTU) analysis (note, “transcript” here refers to a single mRNA splicing isoform). Unlike analyses on the differential abundance of the entire subset of mRNAs transcribed from a given gene, DTU quantifies proportional shifts in the abundance of specific mRNA isoforms relative to other mRNAs transcribed from the same gene.

**Fig. 5:**
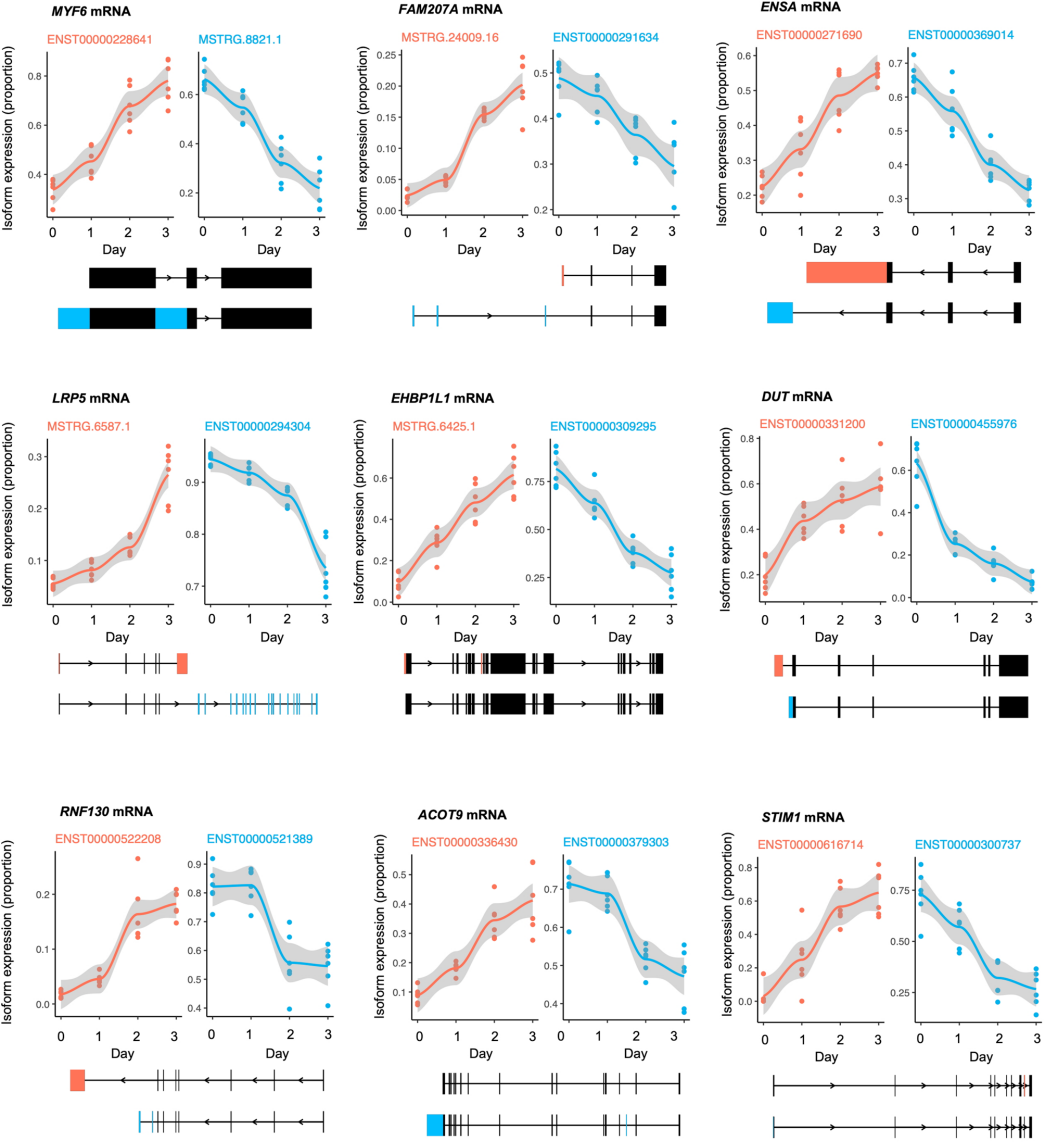
Proportion of alternative spliced mRNA (DTU analysis) during myogenesis. Differential Transcript Usage (DTU) model (days 0 to 3, top 9 by p-adj) for *MYF6*, *FAM207A*, *ENSA*, *LEP5*, *EHBP1L1*, *DUT*, *RNF*130, *ACOT9*, *and STIM1* mRNAs. Each point represents a distinct splicing isoform within a gene, with most genes exhibiting two isoforms, though some display three. The y-axis quantifies the relative abundance of a specific transcript isoform, expressed as a proportion (ranging from 0 to 1) of the total gene expression. The sum of proportions for all isoforms of a given gene at each time point equals 1. Two plots are shown for each gene, highlighting the dynamics of isoform usage. Below each plot, exon schematics, color-coded to distinguish isoforms, depict the structural differences between the splicing variants.

This analysis identified 1,937 events (FDR < 0.05) with notable changes in their splicing variants (Supplementary Material 6, sheet 7), underlying events from 1,200 unique mRNAs. The top 9 most significant differential transcript usage (DTU) events identified from specific mRNAs were: *MYF6* mRNA (encoding a key regulator of muscle development and regeneration), *FAM207A* mRNA (encoding a protein involved in ribosome biogenesis), *ENSA* mRNA (encoding an ATP-sensitive potassium channel), *LRP5* mRNA (encoding a protein implicated in bone metabolism and WNT signaling), *EHBP1L1* mRNA (encoding a protein associated with endocytic trafficking), *DUT* mRNA (encoding a protein involved in nucleotide metabolism), *RNF130* mRNA (encoding a protein component of the ubiquitination cascade), *ACOT9* mRNA (encoding a protein implicated in fatty acid metabolism) and STIM1 mRNA (encoding a protein involved in calcium signaling) (Fig. 5).

**Fig. 6:**
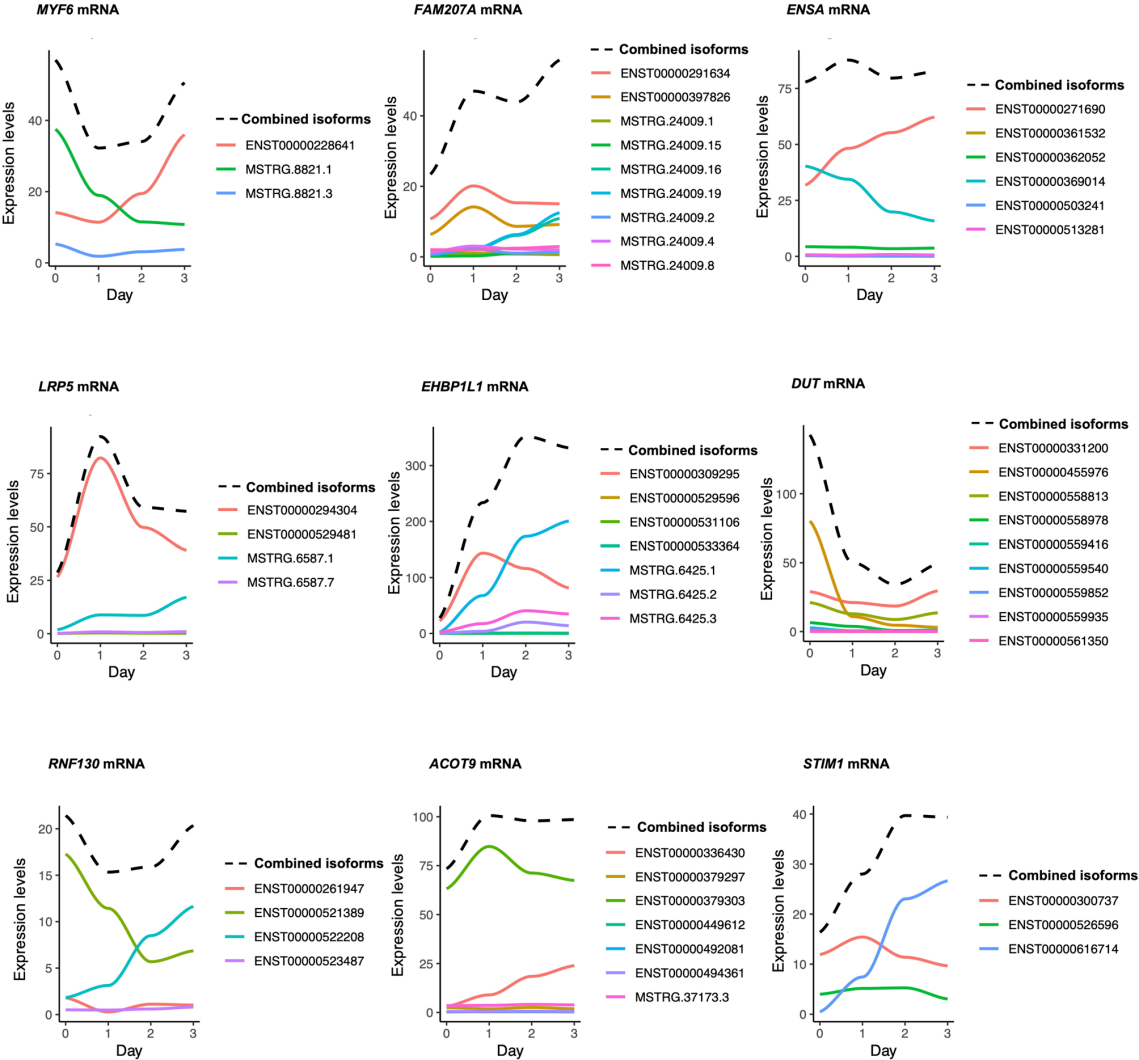
Visualization of splicing isoform expression levels based on DTU analysis. Differential Isoform Expression (DIE) analysis: identification of mRNA splicing variants that significantly influence the transcripts expressed from a given mRNA family. This analysis focused on the top 9 differentially used transcripts (DTU), as ranked by adjusted p-value (see Fig. 5). The solid lines, each in a distinct color, depict individual splicing isoforms. The dotted line represents the combined (total) mRNA expression.

Next, we validated in both AB1167 and AB678 cell lines the expression levels of the two mRNA variants corresponding to *FAM207A* mRNA and *ENSA* mRNA over days 0 to 3 using RT-qPCR analysis (Supplementary Material 9a-b, respectively). A comprehensive overrepresentation analysis (ORA) was conducted on the full set of 1,200 mRNAs associated with the DTU events described above. This analysis revealed significant enrichment in pathways primarily related to muscle physiology and structure. Specifically, we observed overrepresentation of pathways involved in muscle system processes, actin cytoskeleton organization, muscle contraction and its regulation, myofibril assembly, and sarcomere organization (Supplementary Material 10 and Supplementary Material 6, sheet 8). We visualized the expression levels of individual and combined splicing isoforms for the 9 most significant DTU events previously described (Fig. 6). This analysis can be referred to as Differential Isoform Expression (DIE) and confirmed the presence of isoform switches for mRNAs transcribed from five protein-coding mRNA genes (*MYF6*,* ENSA*,* EHBP1L1*,* RNF130*,* and STIM1*). For some mRNAs (*FAM207A* and DUT mRNAs), all isoforms contributed to the changes over time, while for other mRNAs (*LRP5* and *ACOT9* mRNAs), single specific isoforms were almost fully responsible for the collective changes in mRNAs expressed from that gene.

These findings underscore the value of DTU analysis in capturing essential changing splicing isoforms.

### Splicing isoform differentiation dynamics and novel myogenesis-associated variants detection by AI-neural network

**Fig. 7:**
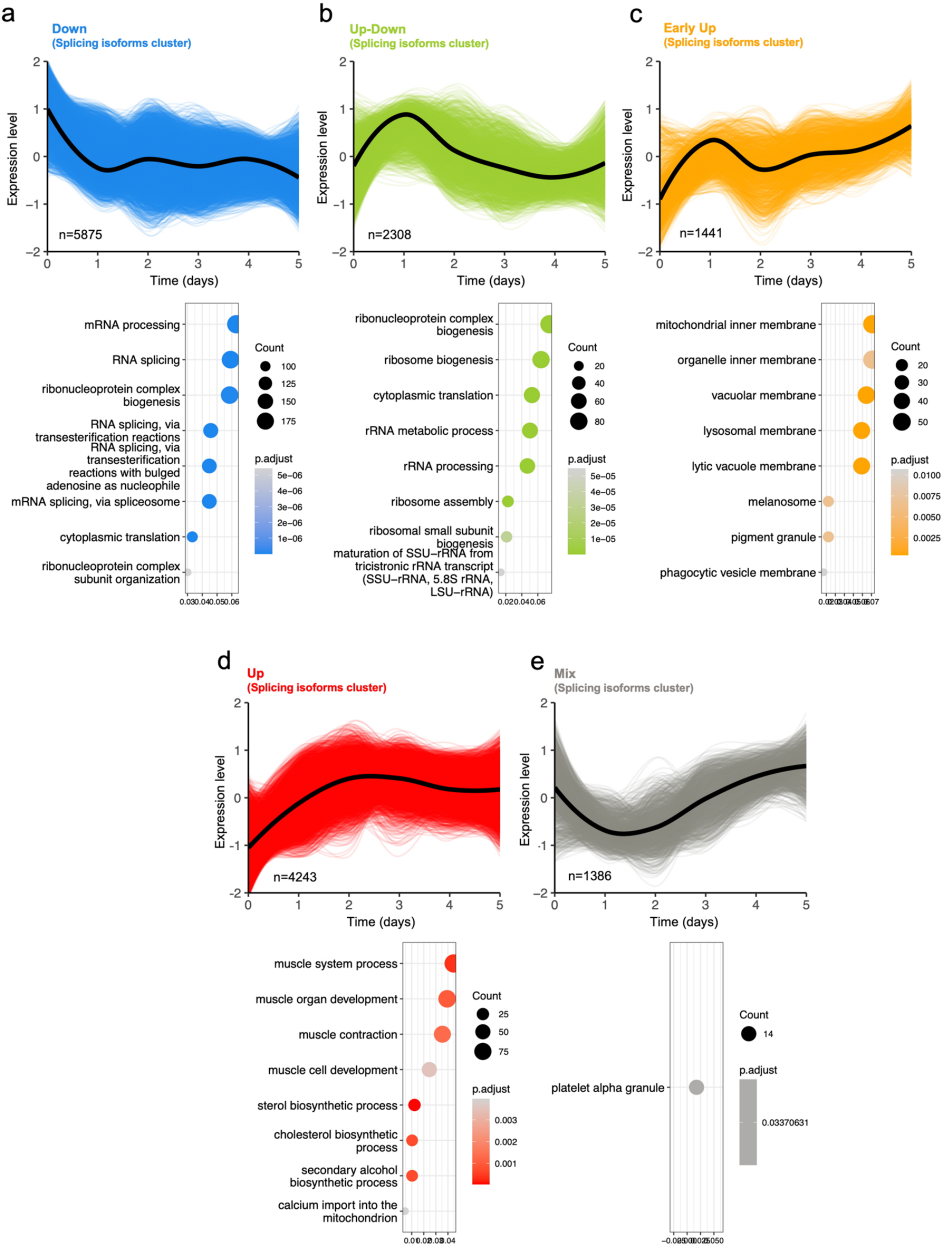
Splicing isoform cluster dynamics during muscle differentiation. Identification of five splicing isoform clusters during full model (0–5 days) myogenesis using a Hierarchical Clustering- Likelihood Ratio Test (HC-LRT) approach. Top panels represent the dynamics of the cluster identified. Bottom panel: Pathway analysis for the clusters identified using Over-Representation Analysis (ORA) representing top 10 pathways associated to myogenesis. For ORA, mRNA family name was used as input for the analysis. **(a)** Down splicing isoform cluster (5875 splicing isoform identified). **(b)** Up-Down splicing isoform cluster (2308 splicing isoform identified). **(c)** Eary Up splicing isoform cluster (1441 splicing isoform identified). **(d)** Up splicing isoform cluster (4243 splicing isoform identified). **(e)** Mix splicing isoform cluster (1386 splicing isoform identified).

To maximize the identification of unique splicing isoforms associated with myogenesis, rather than aggregating all variants mapping to a given mRNA, we employed a non-linear approach using HC-LRT, analogous to the analysis previously conducted at the total mRNA level (Supplementary Material 6, sheet 9). Considering the extensive variability in splicing variants per transcribed gene, ranging from just a few to hundreds, we anticipated finding a greater number of isoform clusters compared to the prior analysis that focused on overall mRNA expression. We identified five distinct isoform clusters associated with myogenesis, each exhibiting a statistical significance of p-adj < 0.01: “Down” (*n* = 5,875, declining initially and then stable, Fig. 7a), “Up-Down” (*n* = 2,308, characterized by initial increase, following decrease, Fig. 7b), “Early-Up” (*n* = 1,441, major initial rise, then slightly increase, Fig. 7c), “Up” (*n* = 4,243, increasing linearly up to day 3, Fig. 7d), and “Mix” (*n* = 1,386, Fig. 7e).

At the mRNA splicing isoform level, overrepresentation analysis (ORA) revealed specific pathway associations for each cluster (Fig. 7a-e, lower panels and Supplementary Material 6, sheet 10). The “Down” cluster was enriched in 162 pathways, with the top 10 related to mRNA splicing. The “Up-Down” cluster showed enrichment in 70 pathways, primarily associated with ribosomal components. The “Early-Up” cluster was involved in 18 pathways linked to lysosomal processes and organelle functions. The “Up” cluster encoded 60 pathways relevant to muscle function, energy metabolism, cholesterol biosynthesis, and cellular organization. Lastly, the “Mix” cluster was associated with a single pathway related to platelet alpha granules. These findings illustrate a dynamic isoform transcriptomic landscape during myogenesis, driving distinct phases of protein expression programs.

To further elucidate the relationship between isoform and RNA clusters, Supplementary Material 11 displays the five isoform clusters (Down, Up-Down, Early Up, Up, Mix), mirroring the order in Fig. 7. Each plot illustrates the distribution of RNAs within these isoform clusters, categorized by their corresponding RNA clusters (Down, Early Down, Early Up, Up, Mix) from Fig. 3, confirming a robust alignment between isoform and RNA clusters. The Down isoform cluster predominantly comprises RNAs from the Down RNA cluster (1,480 RNAs), with smaller contributions from Mix (428 RNAs) and Early Up (256 RNAs). The Up-Down isoform cluster primarily includes RNAs from Early Up (778 RNAs) and Down (601 RNAs), with 238 RNAs from Mix. The Early Up isoform cluster is mainly composed of RNAs from the Early Up RNA cluster (652 RNAs), with 218 RNAs from the Up RNA cluster. The Up isoform cluster is enriched with Early Up RNAs (1,740 RNAs), followed by Up (616 RNAs) and Early Down (438 RNAs). The Mix isoform cluster shows a balanced distribution across all five RNA clusters.

**Fig. 8:**
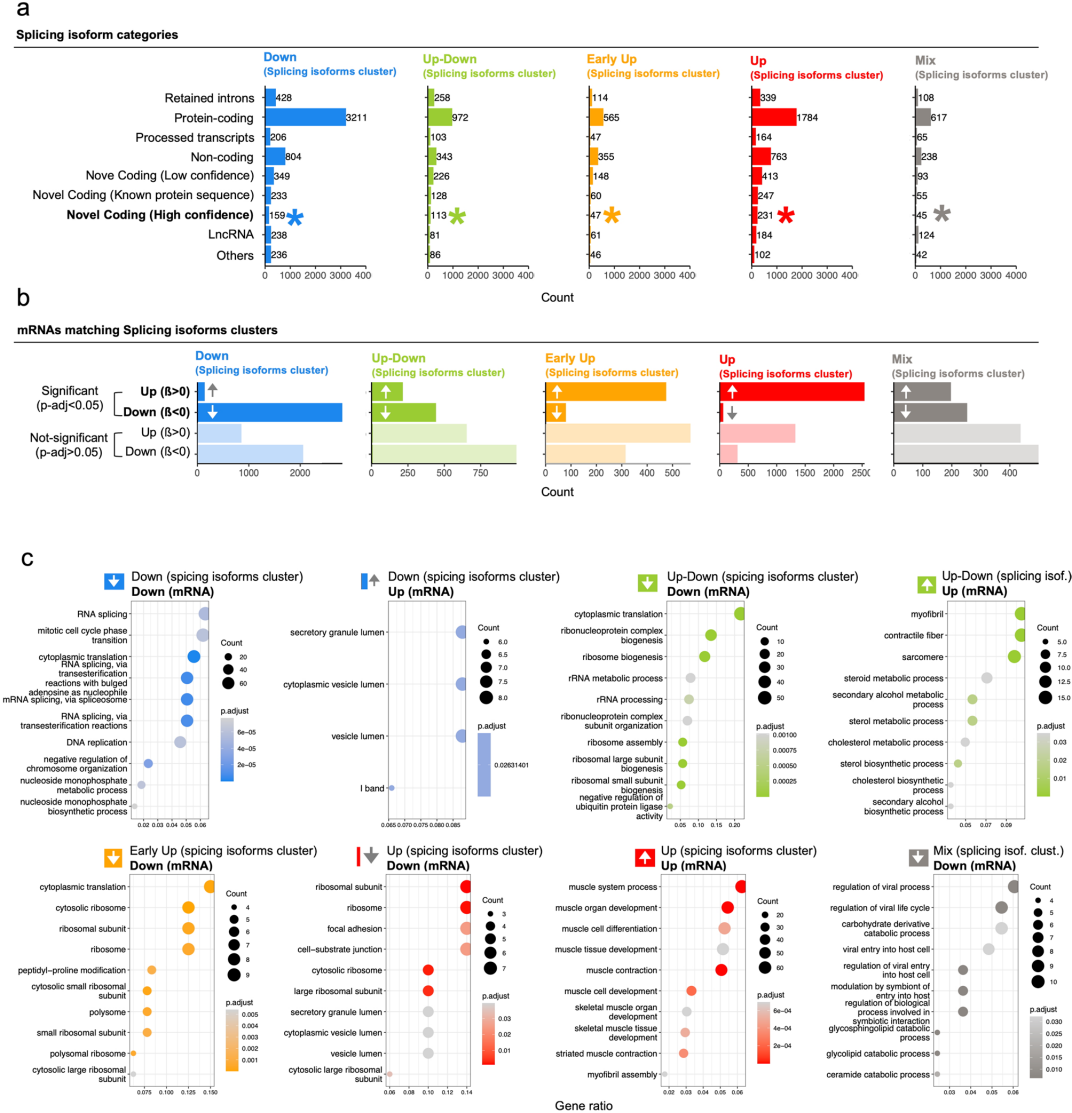
Categorization of splicing variants identified and functional comparison with differential analysis at mRNA family level (pathway analysis). **(a)** Splicing isoform biotype and classification as high or low confidence to be predicted as protein-coding (ESMfold). The category “Novel Coding (Known protein sequence)” are novel isoforms with known protein sequence based on ENSEMBL Database. **(b)** RNA expression levels categories generated from previous Fig. 1 matching splicing isoform cluster in Fig. 7. Light colors represent not-significant mRNA count. **(c)** Pathway analysis for the previous panel **(d)** analysis identified using Over-Representation Analysis (ORA) representing top 10 pathways. We display two ORA plots per splicing isoform cluster related to matching down-regulated and up-regulated mRNA expression levels. ORA results with no significant pathways are omitted.

Next, we quantitatively assessed the distribution and classification of isoform categories within each identified cluster (Fig. 8a and Supplementary Material 6, sheet 11). To identify potential novel proteins expressed from these isoforms, we employed a machine learning algorithm that prioritized high-confidence predictions based on tertiary structure analysis, focusing specifically on those proteins not listed in the ENSEMBL Database. To optimize prediction accuracy, we applied stringent selection criteria, including a predicted local distance difference test score (pLDDT > 0.7) as a threshold for high-confidence inclusion (Fig. 8a, asterisk). This approach enabled us to identify a total of 595 high-confidence novel protein-coding splicing isoforms. Specifically, we found 231 isoforms in the “Up” cluster, 159 in the “Down” cluster, 113 in the “Up-Down” cluster, 47 in the “Early-Up” cluster, and 45 in the “Mix” cluster.

We compared the non-linear splicing isoform analysis to mRNAs exhibiting significant differential expression (Fig. 8b). Unlike Supplementary Material 11, which examined relationships between the five mRNA and the five splicing isoform clusters, the analysis visualized in Fig. 8b analyzed five isoform clusters to determine whether their isoforms enriched mRNAs that were significantly (p-adj < 0.05) up-(ß>0) or downregulated (ß<0), or non-significantly altered (p-adj > 0.05), in the 0–3 day model of Fig. 1d. By cross-referencing RNA family names, we found that significant mRNA abundance (p-adj < 0.05), calculated as the total expression of all splicing isoforms for a given gene, showed an inverse correlation with individual splicing isoforms across all five isoform clusters, with varying strengths of association (see Supplementary Material 6, sheets 12–16 for details).

In Fig. 8c, we performed two comprehensive over-representation analyses (ORA) of biological pathways for each isoform cluster, utilizing the subset of significantly differentially expressed mRNAs identified by the linear regression model from days 0 to 3, as determined in Fig. 8b.

These analyses targeted mRNAs that either showed consistent expression in the same direction or exhibited inverse expression patterns. For example, the “Down” isoform cluster included mRNAs that were downregulated from days 0 to 3, as well as mRNAs that were upregulated during the same period (Supplementary Material 6, sheets 17–24 for all ORA characterized by at least one identified pathway). The consistent expression patterns for the “Down” and “Up” isoform clusters corresponded with previously identified pathways (Fig. 7, lower panels). Specifically, for the “Down” isoform cluster, the association with upregulated mRNA (β > 0, p-adj < 0.05) revealed enriched pathways related to vesicle and granule lumen. In contrast, the “Up-Down” cluster was associated with pathways related to muscle function and metabolism. Interpreting the “Mix” isoform cluster was more complex, and no significant pathways were detected for the “Early-Up” cluster in relation to upregulated mRNA.

The analysis of downregulated mRNA (β < 0, p-adj < 0.05) revealed a significant enrichment of ribosome-related pathways in three distinct isoform clusters: “Early-Up,” “Up,” and “Up-Down,” supporting the notion that the production of ribosomes changes robustly during myogenesis. Taken together, our findings underscore the vigorous regulation of alternatively spliced protein-coding isoforms during myogenic differentiation.

## Discussion

In this study, we used a hybrid RNA-seq strategy integrating short-read and long-read RNA sequencing to investigate transcriptomic changes during myogenic differentiation in two human myoblast cell lines. The hybrid approach was selected based on the findings from the developers of StringTie2, who compared short-read-only, long-read-only, and hybrid methods. Their analysis demonstrated that the hybrid approach outperformed others, achieving superior precision and sensitivity in isoform assembly, resulting in a higher number of accurately assembled isoforms and fewer erroneous ones^44^. This unique strategy led to the identification of 13,853 new un-annotated mRNA splicing isoforms: 6,027 of them contained a splicing site never reported before, while 7,826 contained a splicing event formerly reported. Importantly, after applying the AI-based neural network language model ESMFold, 595 of these isoforms were predicted to encode previously unknown protein variants.

Using non-linear models, we also identified five distinct mRNA clusters generally characterized by lower expression of proteins implicated in cell proliferation and mitotic cell division, as well as increased expression of proteins with function in muscle and metabolic processes, which were also found conserved in myogenesis mouse models. The “early-up” cluster activation (but not the other four clusters), matched the transcriptomic analyses in muscle from peripheral artery disease patients compared to healthy controls. These findings are in keeping with the notion that the muscle damage caused by repeated episodes of ischemia reperfusion in peripheral artery diseases is counteracted by episodes of adult myogenesis.

A deeper transcriptomic level focusing on splicing isoform expression patterns revealed five distinct temporal clusters: ‘down’ (involved in RNA splicing), ‘up-down’ (enriching ribosome biogenesis and metabolism pathways), ‘early-up’ (encoding proteins that participate in mitochondrial inner membrane and lysosomes), ‘up’ (strongly associated to muscle cell differentiation and metabolism), and ‘mix’ (with only a single platelet-related significant pathway). Remarkably, the inverse relationship between splicing activity and mitochondria energy metabolism aligns with the previously described energy-splicing axis of aging^45^.

Integrating individual isoform levels and combined mRNAs mapping to the same gene, we identified three key dynamics of splice variant relative to total mRNA changes: cumulative effects of equally contributing isoforms, single isoform dominance relative to all isoforms, and inverse direction of abundance of one isoform relative to the cumulative abundance of all isoforms. Despite a lower abundance of the cumulative mRNAs transcribed from the same gene, specific and unique splicing isoforms showed high expression during myogenesis with enriched pathways primarily related to muscle processes and ribosomes. These findings provide new insights into the regulation of transcriptomes during myogenesis that could help understand aberrant gene expression patterns in muscle disorders. We have also gained a systematic appreciation for the importance of characterizing individual mRNA isoforms expressed from a given gene relative to cumulative mRNAs expressed from the same gene during myogenesis.

The clusters of expressed cumulative mRNAs and splicing isoforms identified during myogenesis did not mirror those changes associated with muscle during aging or with cardiovascular fitness. While a lack of association with muscle biopsies from individuals in a healthy aging cohort (GESTALT) was anticipated, these analytical approaches could prove more valuable if applied to individuals who are frail or have sarcopenia or cachexia, as they may undergo more active myogenesis to restore lost muscle. Such analyses would be even more useful if the studies were carried out using skeletal muscle biopsies analyzed by methods that captured splicing variant profiles. Our study was based on a comprehensive analysis of splice variants, and we assumed that the mRNAs identified would be translated into proteins, although there is extensive evidence of discordances between mRNA levels and the levels of the presumed encoded protein. This study relies on bioinformatic validation of predicted isoforms without confirming their translation or protein-level activity through proteomic, Western blot, Ribo-seq, or other orthogonal methods, limiting their biological and therapeutic interpretability; future work will explore whether the RNA isoforms identified are translated into proteins or have regulatory functions, and what is the functional role of these proteins or transcripts.

In the future, we aim to investigate to what extent the levels of available energy affect myogenesis and might lead to compensatory mechanisms, including the production of new protein splicing variants, by selectively blocking mitochondrial activity. It is worth noting that this state of limited energy availability caused by low oxygen conditions (hypoxia) may mirror events that occur during fetal development in complicated pregnancies or similar situations characterized by reduced oxygen supply. We will also strive to investigate if the splice variants identified at the transcriptomic level are translated into proteins. Related to this effort, proteomic analysis could validate AI-generated predictions, while metabolomic analysis could confirm pathway activation, providing a more complete understanding of myogenesis. Selective alteration of mitochondrial oxidative phosphorylation aims to detect splicing isoforms conferring mitochondrial resilience, as previously hypothesized^45^. This analysis can be carried out using specific inhibitors of the electron transport chain complexes I and II, as well as ATP synthase or complex V. In mouse models, modulation of molecular components of complex V, such as ATPIF1, allows analysis of the activation of mitochondrial target splicing variants.

## Conclusions

Our study has characterized the transcriptomic and mRNA splicing variant changes during myogenesis, suggesting the existence of conserved mechanisms across species and human disease states. The comprehensive catalog of myogenesis-associated splicing isoforms reported here is a valuable resource for developing RNA therapies targeting muscle regeneration and age-related muscle conditions.

## Supplementary Information

Below is the link to the electronic supplementary material.

Supplementary Material 1

Supplementary Material 2

Supplementary Material 3

Supplementary Material 4

Supplementary Material 5

Supplementary Material 6

Supplementary Material 7

Supplementary Material 8

Supplementary Material 9

Supplementary Material 10

Supplementary Material 11

## Data Availability

Data generated and analyzed are available in the GEO repository, under the accession number GSE287954. The R code is available on Github ( https://github.com/niairpnih/splicingmyogenesis2025 ). For any additional request, please contact directly the corresponding author [ferruccilu@grc.nia.nih.gov] (mailto: ferruccilu@grc.nia.nih.gov) .
