# Supplementary material for: In-vitro human myogenesis model reveals novel mRNA alternative splicing isoforms
Source: Sci Rep. 2025 Oct 1;15:34273. doi: 10.1038/s41598-025-16523-2 (PMC12489129; doi:10.1038/s41598-025-16523-2)
Supplement: Supplementary file 1 — Supplementary Material 1 [file 41598_2025_16523_MOESM1_ESM.pdf]

mRNA validated	Primer	Oligo sequence
ADA	Forward	CTGCTGAACGTCATTGGCATGG
ADA	Reverse	GGCGATCCTTTTGATAGCCTCC
ALOX5AP	Reverse	AGACCAGAGCACAGCGAGGAAA
ALOZ5AP	Forward	AAGTGGAGCACGAAAGCAGGAC
ATOH8	Forward	CATCCCAGGCAACAAACAGC
ATOH8	Reverse	AGCAAGACGAGAACAACGGT
CENPS	Forward	GGCTAGGGTGCTTTTTGTGC
CENPS	Reverse	TCTGGAGTGAGACCTGGGAG
CENPW	Forward	AGAAGAGCAGAGGTTAGAAGTCA
CENPW	Reverse	TCTGTTACCACCCAAAAGAATGC
CSRP3	Forward	CACTGCGAAGTTTGGAGAGTCC
CSRP3	Reverse	AGCGGAAACAGGTCTTGTGCCA
ESPL1	Forward	ATCTCTGTCAGTCGGACCTGCA
ESPL1	Reverse	CAGGTGGACCTTCTTCACAGAG
GAPDH	Forward	CTCTGCTCCTCCTGTTCGAC
GAPDH	Reverse	ACGACCAAATCCGTTGACTC
GINS2	Forward	AGCCAAACTCCGAGTGTCTGCT
GINS2	Reverse	CTTGTGTGAGGAAAGTCCCGCT
LINC02709	Forward	GGTTGTCTGCAGAGTGAGAT
LINC02709	Reverse	TGGAAAACTGAGAAAAAGCAGGT
MYH2	Forward	GGAGGACAAAGTCAACACCCTG
MYH2	Reverse	GCCCTTTCTAGGTCCATGCGAA
MYL2	Forward	GCAGTGCTGGGTCCTTTCCA
MYL2R	Reverse	TGAAGGCCTCCTTAAATTCCTGG
MYMX	Forward	CCACAGACATGCCTCTCCAC
MYMX	Reverse	AGGAGTGGTCTTTGGGAGGA
MYOM1	Forward	TGTCAAGGCTGTCAGTGAGGAG
MYOM1	Reverse	GAAGCATTCGCTTACTGCGGAG
MYOM2	Forward	CTACCTGGACAAGCGTGAAGTTC
MYOM2	Reverse	CGTAGAGTGAGCCTTCCGTCAA
MYOM3	Forward	GCCGATGAAGACATCTCCGCAA
MYOM3	Reverse	CCAGGATGTCAATGTTCCAGCC
SPC24	Forward	GGGATTATGAGTGTGAGCCAGG
SPC24	Reverse	ACTCCAGAGGTAGTCGCTGATG
SVIL	Forward	CTCGCCTCAGAGTCCGTAAC
SVIL	Reverse	GGGGACCCTATCAAAGGCTG
TSPAN7	Forward	CTCATCGGAACTGGCACCACTA
TSPAN7	Reverse	CCTGAAATGCCAGCTACGAGCT
VIM	Forward	AGGCAAAGCAGGAGTCCACTGA
VIM	Reverse	ATCTGGCGTTCCAGGGACTCAT
MSTRG.4237.1	Forward	GAGTATCAGAGTGAAACAAAACC
MSTRG.4237.1	Reverse	GTTGACAGGTAGCTATTAAGC
MSTRG.12012.1(RT)	Forward	GTGATCTTGAAAGTGTCTTCG
MSTRG.12012.1	Reverse	TTACCGATTACGTTTCGTCG
MSTRG.21234.1(RT)	Forward	CAAAGTGACACAATCGGTCC
MSTRG.21234.1	Reverse	CCGGACATTAGGGTCGTGAAGCC

Supplementary Material 1. Oligonucleotide primers used for RT-qPCR validation
