# Supplementary material for: In-vitro human myogenesis model reveals novel mRNA alternative splicing isoforms
Source: Sci Rep. 2025 Oct 1;15:34273. doi: 10.1038/s41598-025-16523-2 (PMC12489129; doi:10.1038/s41598-025-16523-2)
Supplement: Supplementary file 2 — Supplementary Material 2 [file 41598_2025_16523_MOESM2_ESM.pdf]

Supplementary Material 2. Software versions used in Rstudio to generate analysis

```
─ Session info ───────────────────────────────────────────────────────────────
 setting  value
 version  R version 4.2.3 (2023-03-15)
 os       Ubuntu 20.04.3 LTS
 system   x86_64, linux-gnu
 ui       RStudio
 language (EN)
 collate  C.UTF-8
 ctype    C.UTF-8
 tz       Etc/UTC
 date     2025-01-16
 rstudio  2022.07.2+576 Spotted Wakerobin (server)
 pandoc   2.19.2 @ /usr/lib/rstudio-server/bin/quarto/bin/tools/ (via rmarkdown)

─ Packages ───────────────────────────────────────────────────────────────────
 package              * version    date (UTC) lib source
 abind                  1.4-5      2016-07-21 [1] CRAN (R 4.2.3)
 annotate               1.76.0     2022-11-01 [1] Bioconductor
 AnnotationDbi        * 1.60.2     2023-03-10 [1] Bioconductor
 ape                    5.8        2024-04-11 [1] CRAN (R 4.2.3)
 apeglm               * 1.20.0     2022-11-01 [1] Bioconductor
 aplot                  0.2.2      2023-10-06 [1] CRAN (R 4.2.3)
 babelgene              22.9       2022-09-29 [1] CRAN (R 4.2.1)
 backports              1.4.1      2021-12-13 [1] CRAN (R 4.2.1)
 base64enc              0.1-3      2015-07-28 [1] CRAN (R 4.2.1)
 bbmle                  1.0.25.1   2023-12-09 [1] CRAN (R 4.2.3)
 bdsmatrix              1.3-7      2024-03-02 [1] CRAN (R 4.2.3)
 Biobase              * 2.58.0     2022-11-01 [1] Bioconductor
 BiocFileCache          2.6.1      2023-02-17 [1] Bioconductor
 BiocGenerics         * 0.44.0     2022-11-01 [1] Bioconductor
 BiocIO                 1.8.0      2022-11-01 [1] Bioconductor
 BiocParallel         * 1.32.6     2023-03-17 [1] Bioconductor
 biomaRt                2.54.1     2023-03-20 [1] Bioconductor
 Biostrings           * 2.66.0     2022-11-01 [1] Bioconductor
 bit                    4.0.5      2022-11-15 [1] CRAN (R 4.2.1)
 bit64                  4.0.5      2020-08-30 [1] CRAN (R 4.2.1)
 bitops                 1.0-7      2021-04-24 [1] CRAN (R 4.2.1)
 blob                   1.2.4      2023-03-17 [1] CRAN (R 4.2.3)
 broom                * 1.0.5      2023-06-09 [1] CRAN (R 4.2.3)
 cachem                 1.0.8      2023-05-01 [1] CRAN (R 4.2.3)
 car                    3.1-2      2023-03-30 [1] CRAN (R 4.2.3)
 carData                3.0-5      2022-01-06 [1] CRAN (R 4.2.3)
 cellranger             1.1.0      2016-07-27 [1] CRAN (R 4.2.1)
 checkmate              2.3.1      2023-12-04 [1] CRAN (R 4.2.3)
 cli                    3.6.2      2023-12-11 [1] CRAN (R 4.2.3)
 cluster                2.1.4      2022-08-22 [4] CRAN (R 4.2.1)
 clusterProfiler      * 4.6.2      2023-03-05 [1] Bioconductor
 coda                   0.19-4.1   2024-01-31 [1] CRAN (R 4.2.3)
 codetools              0.2-20     2024-03-31 [1] CRAN (R 4.2.3)
 colorspace             2.1-0      2023-01-23 [1] CRAN (R 4.2.3)
 commonmark             1.9.1      2024-01-30 [1] CRAN (R 4.2.3)
 cowplot                1.1.3      2024-01-22 [1] CRAN (R 4.2.3)
```

```
crayon                 1.5.2      2022-09-29 [1] CRAN (R 4.2.1)
curl                   5.2.1      2024-03-01 [1] CRAN (R 4.2.3)
data.table           * 1.15.4     2024-03-30 [1] CRAN (R 4.2.3)
DBI                    1.2.2      2024-02-16 [1] CRAN (R 4.2.3)
dbplyr                 2.5.0      2024-03-19 [1] CRAN (R 4.2.3)
DelayedArray           0.24.0     2022-11-01 [1] Bioconductor
DESeq2               * 1.38.3     2023-01-19 [1] Bioconductor
DEXSeq               * 1.44.0     2022-11-01 [1] Bioconductor
digest                 0.6.35     2024-03-11 [1] CRAN (R 4.2.3)
DOSE                   3.24.2     2022-11-21 [1] Bioconductor
downloader             0.4        2015-07-09 [1] CRAN (R 4.2.1)
dplyr                * 1.1.4      2023-11-17 [1] CRAN (R 4.2.3)
drc                  * 3.0-1      2016-08-30 [1] CRAN (R 4.2.3)
DRIMSeq              * 1.26.0     2022-11-01 [1] Bioconductor
edgeR                * 3.40.2     2023-01-19 [1] Bioconductor
emdbook                1.3.13     2023-07-03 [1] CRAN (R 4.2.3)
EnhancedVolcano      * 1.16.0     2022-11-01 [1] Bioconductor
enrichplot             1.18.4     2023-04-03 [1] Bioconductor
eulerr               * 7.0.2      2024-03-28 [1] CRAN (R 4.2.3)
evaluate               0.23       2023-11-01 [1] CRAN (R 4.2.3)
fansi                  1.0.6      2023-12-08 [1] CRAN (R 4.2.3)
farver                 2.1.1      2022-07-06 [1] CRAN (R 4.2.1)
fastmap                1.1.1      2023-02-24 [1] CRAN (R 4.2.3)
fastmatch              1.1-4      2023-08-18 [1] CRAN (R 4.2.3)
fgsea                  1.24.0     2022-11-01 [1] Bioconductor
filelock               1.0.3      2023-12-11 [1] CRAN (R 4.2.3)
forcats              * 1.0.0      2023-01-29 [1] CRAN (R 4.2.3)
foreign                0.8-86     2023-11-28 [1] CRAN (R 4.2.3)
Formula                1.2-5      2023-02-24 [1] CRAN (R 4.2.3)
fs                     1.6.3      2023-07-20 [1] CRAN (R 4.2.3)
genefilter             1.80.3     2023-01-19 [1] Bioconductor
geneplotter            1.76.0     2022-11-01 [1] Bioconductor
generics               0.1.3      2022-07-05 [1] CRAN (R 4.2.1)
GenomeInfoDb         * 1.34.9     2023-02-02 [1] Bioconductor
GenomeInfoDbData       1.2.9      2023-06-21 [1] Bioconductor
GenomicAlignments      1.34.1     2023-03-09 [1] Bioconductor
GenomicFeatures      * 1.50.4     2023-01-24 [1] Bioconductor
GenomicRanges        * 1.50.2     2022-12-16 [1] Bioconductor
ggbreak              * 0.1.2      2023-06-26 [1] CRAN (R 4.2.3)
ggforce                0.4.2      2024-02-19 [1] CRAN (R 4.2.3)
ggfun                  0.1.4      2024-01-19 [1] CRAN (R 4.2.3)
ggplot2              * 3.5.1      2024-04-23 [1] CRAN (R 4.2.3)
ggplotify              0.1.2      2023-08-09 [1] CRAN (R 4.2.3)
ggraph                 2.2.1      2024-03-07 [1] CRAN (R 4.2.3)
ggrepel              * 0.9.5      2024-01-10 [1] CRAN (R 4.2.3)
ggtranscript         * 0.99.9     2022-10-06 [1] Github (dzhang32/ggtranscript@b99da0d)
ggtree                 3.6.2      2022-11-10 [1] Bioconductor
ggVennDiagram        * 1.5.2      2024-02-20 [1] CRAN (R 4.2.3)
glue                   1.7.0      2024-01-09 [1] CRAN (R 4.2.3)
GO.db                  3.16.0     2023-06-21 [1] Bioconductor
GOSemSim               2.24.0     2022-11-01 [1] Bioconductor
graphlayouts           1.1.1      2024-03-09 [1] CRAN (R 4.2.3)
gridExtra              2.3        2017-09-09 [1] CRAN (R 4.2.1)
gridGraphics           0.5-1      2020-12-13 [1] CRAN (R 4.2.1)
gson                   0.1.0      2023-03-07 [1] CRAN (R 4.2.3)
gt                   * 0.10.1     2024-01-17 [1] CRAN (R 4.2.3)
gtable                 0.3.5      2024-04-22 [1] CRAN (R 4.2.3)
gtools                 3.9.5      2023-11-20 [1] CRAN (R 4.2.3)
HDO.db                 0.99.1     2023-06-21 [1] Bioconductor
Hmisc                  5.1-2      2024-03-11 [1] CRAN (R 4.2.3)
hms                    1.1.3      2023-03-21 [1] CRAN (R 4.2.3)
```

```
 htmlTable             2.4.2       2023-10-29 [1] CRAN (R 4.2.3)
 htmltools             0.5.8.1     2024-04-04 [1] CRAN (R 4.2.3)
 htmlwidgets           1.6.4       2023-12-06 [1] CRAN (R 4.2.3)
 httr                  1.4.7       2023-08-15 [1] CRAN (R 4.2.3)
 hwriter               1.3.2.1     2022-04-08 [1] CRAN (R 4.2.1)
 igraph              * 2.0.3       2024-03-13 [1] CRAN (R 4.2.3)
 IRanges             * 2.32.0      2022-11-01 [1] Bioconductor
 jsonlite              1.8.8       2023-12-04 [1] CRAN (R 4.2.3)
 KEGGREST              1.38.0      2022-11-01 [1] Bioconductor
 knitr                 1.46        2024-04-06 [1] CRAN (R 4.2.3)
 labeling              0.4.3       2023-08-29 [1] CRAN (R 4.2.3)
 lattice               0.22-6      2024-03-20 [1] CRAN (R 4.2.3)
 lazyeval              0.2.2       2019-03-15 [1] CRAN (R 4.2.1)
 lifecycle             1.0.4       2023-11-07 [1] CRAN (R 4.2.3)
 limma               * 3.54.2      2023-02-28 [1] Bioconductor
 locfit                1.5-9.9     2024-03-01 [1] CRAN (R 4.2.3)
 lubridate           * 1.9.3       2023-09-27 [1] CRAN (R 4.2.3)
 magrittr              2.0.3       2022-03-30 [1] CRAN (R 4.2.1)
 markdown              1.12        2023-12-06 [1] CRAN (R 4.2.3)
 MASS                * 7.3-60.0.1  2024-01-13 [1] CRAN (R 4.2.3)
 Matrix                1.6-5       2024-01-11 [1] CRAN (R 4.2.3)
 MatrixGenerics      * 1.10.0      2022-11-01 [1] Bioconductor
 matrixStats         * 1.3.0       2024-04-11 [1] CRAN (R 4.2.3)
 memoise               2.0.1       2021-11-26 [1] CRAN (R 4.2.1)
 mgcv                  1.9-1       2023-12-21 [1] CRAN (R 4.2.3)
 msigdbr             * 7.5.1       2022-03-30 [1] CRAN (R 4.2.1)
 multcomp              1.4-25      2023-06-20 [1] CRAN (R 4.2.3)
 munsell               0.5.1       2024-04-01 [1] CRAN (R 4.2.3)
 mvtnorm               1.2-4       2023-11-27 [1] CRAN (R 4.2.3)
 nlme                  3.1-164     2023-11-27 [1] CRAN (R 4.2.3)
 nnet                  7.3-19      2023-05-03 [1] CRAN (R 4.2.3)
 numDeriv              2016.8-1.1  2019-06-06 [1] CRAN (R 4.2.1)
 org.Hs.eg.db        * 3.16.0      2023-06-21 [1] Bioconductor
 patchwork           * 1.2.0       2024-01-08 [1] CRAN (R 4.2.3)
 pheatmap            * 1.0.12      2019-01-04 [1] CRAN (R 4.2.1)
 pillar                1.9.0       2023-03-22 [1] CRAN (R 4.2.3)
 pkgconfig             2.0.3       2019-09-22 [1] CRAN (R 4.2.1)
 plotrix               3.8-4       2023-11-10 [1] CRAN (R 4.2.3)
 plyr                  1.8.9       2023-10-02 [1] CRAN (R 4.2.3)
 png                   0.1-8       2022-11-29 [1] CRAN (R 4.2.1)
 polyclip              1.10-6      2023-09-27 [1] CRAN (R 4.2.3)
 prettyunits           1.2.0       2023-09-24 [1] CRAN (R 4.2.3)
 progress              1.2.3       2023-12-06 [1] CRAN (R 4.2.3)
 purrr               * 1.0.2       2023-08-10 [1] CRAN (R 4.2.3)
 qvalue                2.30.0      2022-11-01 [1] Bioconductor
 R6                    2.5.1       2021-08-19 [1] CRAN (R 4.2.1)
 rappdirs              0.3.3       2021-01-31 [1] CRAN (R 4.2.1)
 RColorBrewer        * 1.1-3       2022-04-03 [1] CRAN (R 4.2.1)
 Rcpp                  1.0.12      2024-01-09 [1] CRAN (R 4.2.3)
 RCurl                 1.98-1.14   2024-01-09 [1] CRAN (R 4.2.3)
 readr               * 2.1.5       2024-01-10 [1] CRAN (R 4.2.3)
 readxl              * 1.4.3       2023-07-06 [1] CRAN (R 4.2.3)
 reshape2              1.4.4       2020-04-09 [1] CRAN (R 4.2.1)
 restfulr              0.0.15      2022-06-16 [1] CRAN (R 4.2.1)
 rjson                 0.2.21      2022-01-09 [1] CRAN (R 4.2.1)
 rlang                 1.1.3       2024-01-10 [1] CRAN (R 4.2.3)
 rmarkdown             2.26        2024-03-05 [1] CRAN (R 4.2.3)
 rpart                 4.1.23      2023-12-05 [1] CRAN (R 4.2.3)
 Rsamtools             2.14.0      2022-11-01 [1] Bioconductor
 RSQLite               2.3.6       2024-03-31 [1] CRAN (R 4.2.3)
 rstudioapi            0.16.0      2024-03-24 [1] CRAN (R 4.2.3)
```

```
 rtracklayer            1.58.0      2022-11-01 [1] Bioconductor
 S4Vectors            * 0.36.2      2023-02-26 [1] Bioconductor
 sandwich               3.1-0       2023-12-11 [1] CRAN (R 4.2.3)
 sass                   0.4.9       2024-03-15 [1] CRAN (R 4.2.3)
 scales                 1.3.0       2023-11-28 [1] CRAN (R 4.2.3)
 scatterpie             0.2.2       2024-04-03 [1] CRAN (R 4.2.3)
 sessioninfo          * 1.2.2       2021-12-06 [1] CRAN (R 4.2.3)
 shadowtext             0.1.3       2024-01-19 [1] CRAN (R 4.2.3)
 stageR               * 1.20.0      2022-11-01 [1] Bioconductor
 statmod                1.5.0       2023-01-06 [1] CRAN (R 4.2.3)
 stringi                1.8.3       2023-12-11 [1] CRAN (R 4.2.3)
 stringr              * 1.5.1       2023-11-14 [1] CRAN (R 4.2.3)
 SummarizedExperiment * 1.28.0      2022-11-01 [1] Bioconductor
 survival               3.5-8       2024-02-14 [1] CRAN (R 4.2.3)
 TH.data                1.1-2       2023-04-17 [1] CRAN (R 4.2.3)
 tibble               * 3.2.1       2023-03-20 [1] CRAN (R 4.2.3)
 tidygraph              1.3.1       2024-01-30 [1] CRAN (R 4.2.3)
 tidyr                * 1.3.1       2024-01-24 [1] CRAN (R 4.2.3)
 tidyselect             1.2.1       2024-03-11 [1] CRAN (R 4.2.3)
 tidytree               0.4.6       2023-12-12 [1] CRAN (R 4.2.3)
 tidyverse            * 2.0.0       2023-02-22 [1] CRAN (R 4.2.3)
 timechange             0.3.0       2024-01-18 [1] CRAN (R 4.2.3)
 treeio                 1.22.0      2022-11-01 [1] Bioconductor
 tweenr                 2.0.3       2024-02-26 [1] CRAN (R 4.2.3)
 tximport             * 1.26.1      2022-12-16 [1] Bioconductor
 tzdb                   0.4.0       2023-05-12 [1] CRAN (R 4.2.3)
 UpSetR                 1.4.0       2019-05-22 [1] CRAN (R 4.2.3)
 utf8                   1.2.4       2023-10-22 [1] CRAN (R 4.2.3)
 vctrs                  0.6.5       2023-12-01 [1] CRAN (R 4.2.3)
 viridis              * 0.6.5       2024-01-29 [1] CRAN (R 4.2.3)
 viridisLite          * 0.4.2       2023-05-02 [1] CRAN (R 4.2.3)
 vroom                  1.6.5       2023-12-05 [1] CRAN (R 4.2.3)
 withr                  3.0.0       2024-01-16 [1] CRAN (R 4.2.3)
 xfun                   0.43        2024-03-25 [1] CRAN (R 4.2.3)
 XML                    3.99-0.16.1 2024-01-22 [1] CRAN (R 4.2.3)
 xml2                   1.3.6       2023-12-04 [1] CRAN (R 4.2.3)
 xtable                 1.8-4       2019-04-21 [1] CRAN (R 4.2.1)
 XVector              * 0.38.0      2022-11-01 [1] Bioconductor
 yaml                   2.3.8       2023-12-11 [1] CRAN (R 4.2.3)
 yulab.utils            0.1.4       2024-01-28 [1] CRAN (R 4.2.3)
 zlibbioc               1.44.0      2022-11-01 [1] Bioconductor
 zoo                    1.8-12      2023-04-13 [1] CRAN (R 4.2.3)
```
