# Supplementary figures and images for: In-vitro human myogenesis model reveals novel mRNA alternative splicing isoforms
Source: Sci Rep. 2025 Oct 1;15:34273. doi: 10.1038/s41598-025-16523-2 (PMC12489129; doi:10.1038/s41598-025-16523-2)

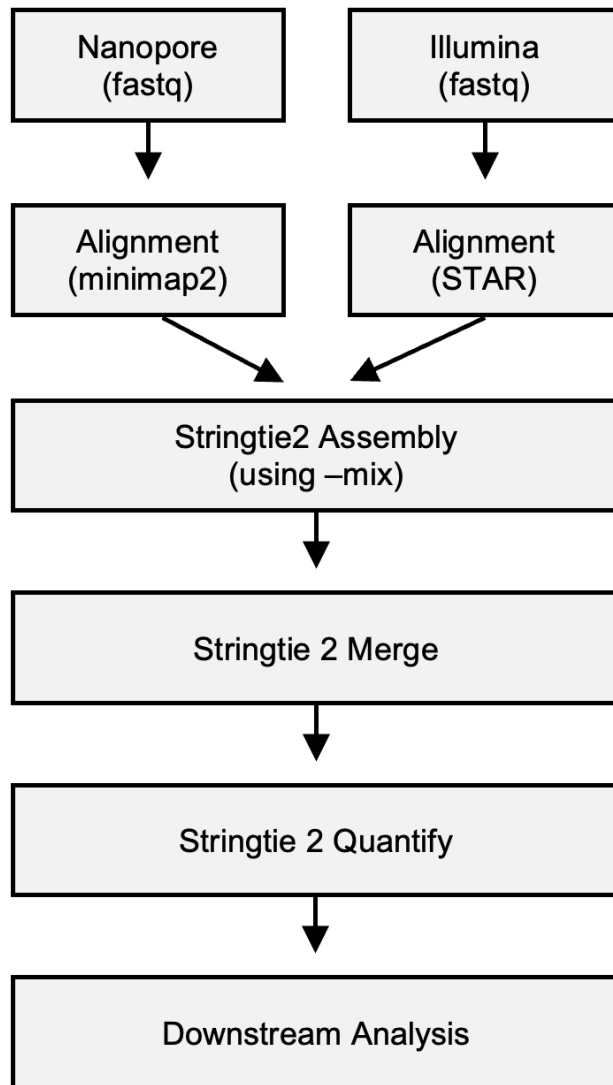

**Supplementary Material 3.** Flow diagram of bioinformatic tools used in this study

Supplement: Supplementary file 3 — Supplementary Material 3 [file 41598_2025_16523_MOESM3_ESM.pdf]
