# Supplementary material for: In-vitro human myogenesis model reveals novel mRNA alternative splicing isoforms
Source: Sci Rep. 2025 Oct 1;15:34273. doi: 10.1038/s41598-025-16523-2 (PMC12489129; doi:10.1038/s41598-025-16523-2)
Supplement: Supplementary file 5 — Supplementary Material 5 [file 41598_2025_16523_MOESM5_ESM.pdf]

a

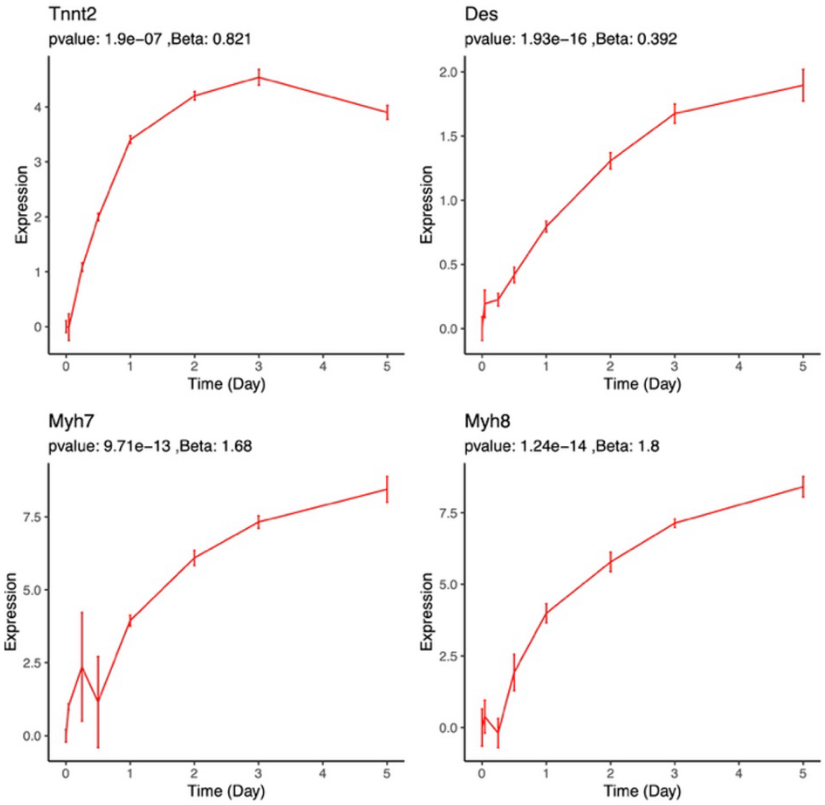

Tnnt2
pvalue: 1.9e−07 ,Beta: 0.821
Des
pvalue: 1.93e−16 ,Beta: 0.392
Myh7
pvalue: 9.71e−13 ,Beta: 1.68
Myh8
pvalue: 1.24e−14 ,Beta: 1.8
Expression
Time (Day)

b

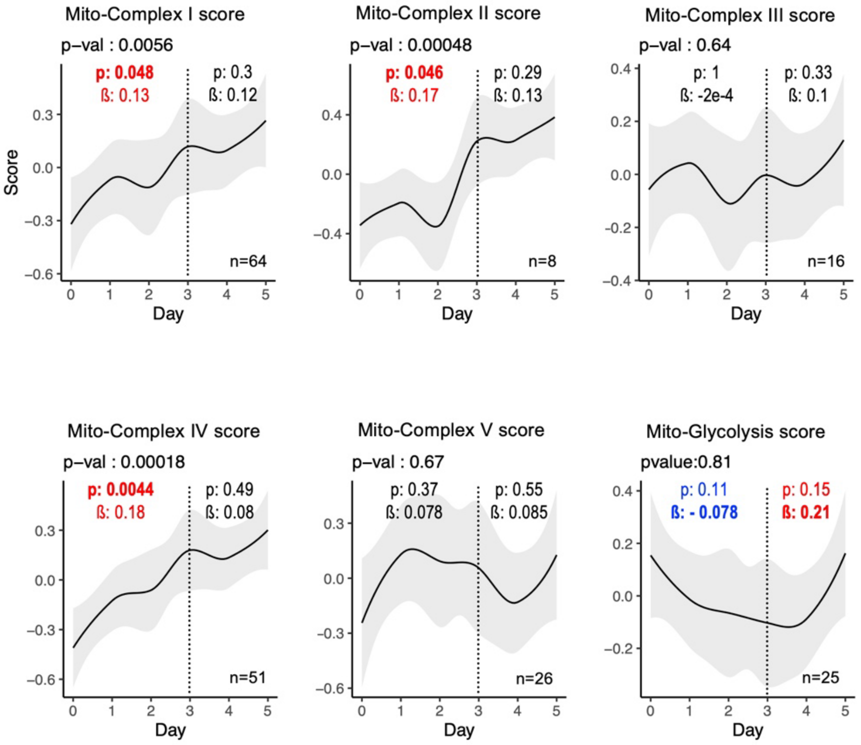

Mito-Complex I score
p−val : 0.0056
p: 0.048
ß: 0.13
p: 0.3
ß: 0.12
n=64
Mito-Complex II score
p−val : 0.00048
p: 0.046
ß: 0.17
p: 0.29
ß: 0.13
n=8
Mito-Complex III score
p−val : 0.64
p: 1
ß: -2e-4
p: 0.33
ß: 0.1
n=16
Mito-Complex IV score
p−val : 0.00018
p: 0.0044
ß: 0.18
p: 0.49
ß: 0.08
n=51
Mito-Complex V score
p−val : 0.67
p: 0.37
ß: 0.078
p: 0.55
ß: 0.085
n=26
Mito-Glycolysis score
pvalue:0.81
p: 0.11
ß: - 0.078
p: 0.15
ß: 0.21
n=25
Score
Day

Supplementary Material 5. Proteomic muscle marker of differentiation from another study dataset and mitochondrial transcriptomic score dynamics during human muscle differentiation. a) Protein expression of four myogenesis markers from an in-vitro mouse model study (Xiao et al., 2022). Time points in the x-axis: 0, 1, 6, 12 hours, then 1, 2, 3, 5 days (day 4 is not present). P-value of linear regression and relative beta value is provided. b) Scores were derived from the MitoCarta 3.0 Dataset over days 0 to 5 of differentiation, with subtitles showing p-values associated with each score. Early (days 0 to 3) and late (days 3 to 5) phases are analyzed separately. Blue color for p represents negative trends, while red indicates positive trends.

Xiao, D., Caldow, M., Kim, H. J., Blazev, R., Koopman, R., Manandi, D., Parker, B. L., & Yang, P. (2022). Time-resolved phosphoproteome and proteome analysis reveals kinase signaling on master transcription factors during myogenesis. *iScience*, *25*(6), 104489. https://doi.org/10.1016/j.isci.2022.104489
