# Supplementary material for: In-vitro human myogenesis model reveals novel mRNA alternative splicing isoforms
Source: Sci Rep. 2025 Oct 1;15:34273. doi: 10.1038/s41598-025-16523-2 (PMC12489129; doi:10.1038/s41598-025-16523-2)
Supplement: Supplementary file 7 — Supplementary Material 7 [file 41598_2025_16523_MOESM7_ESM.pdf]

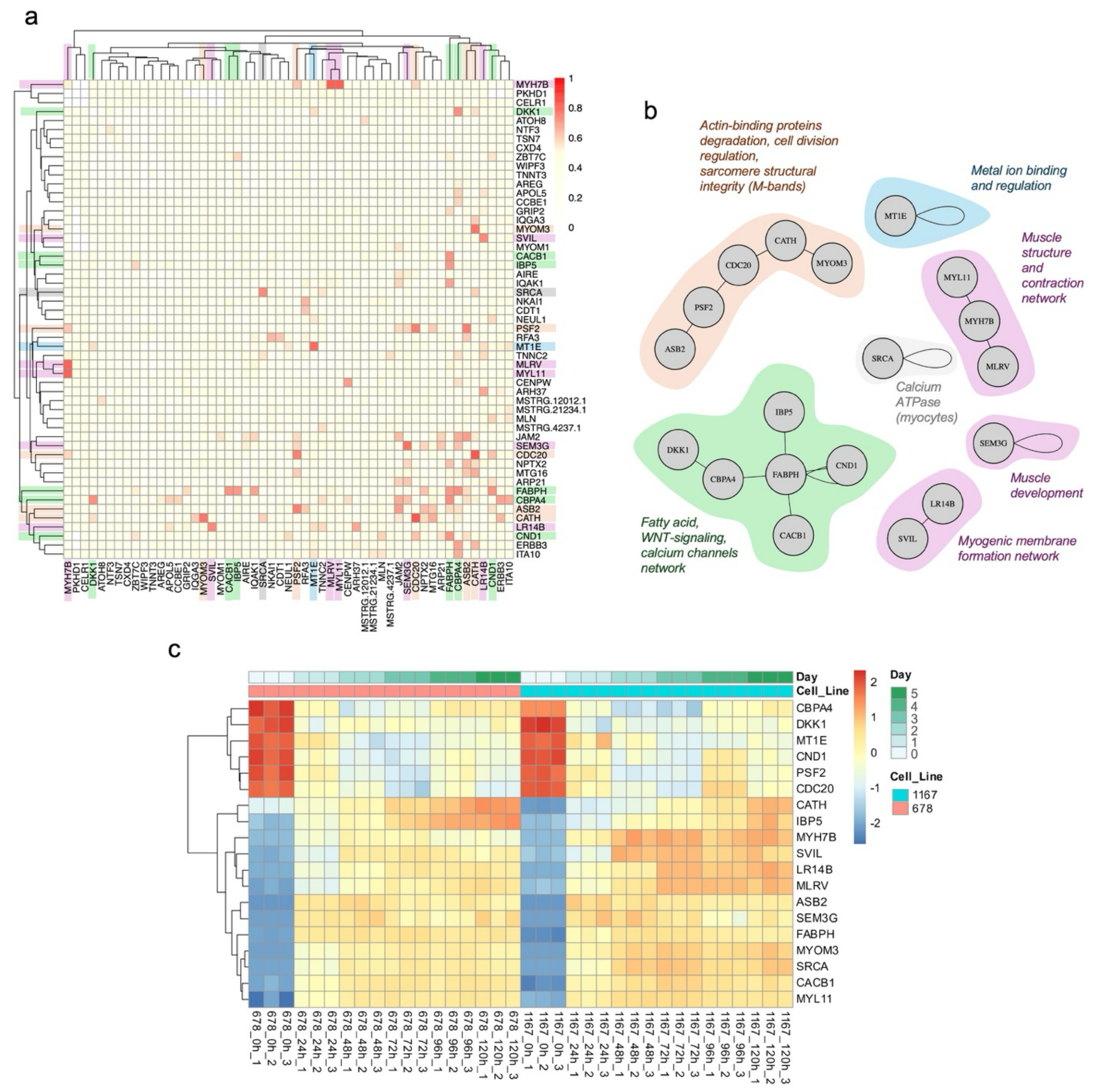

Supplementary Material 7. Protein Interaction Network in Myogenesis. a) Heatmap showing cross-correlation of interaction strengths between the 19 proteins. Color intensity represents the strength of predicted interactions based on AlphaPulldown analysis (ipTM score>0.7). b) Representative visualization of protein-protein interactions among 19 key proteins identified in 7 clusters during myogenesis. Nodes represent proteins, edges indicate predicted interactions, and node colors denote functional clusters. c) Heatmap representing RNA expression of the 19 key items identified across myogenesis. Days, cell lines and expression are represented with color-coding in legend
