# Supplementary material for: In-vitro human myogenesis model reveals novel mRNA alternative splicing isoforms
Source: Sci Rep. 2025 Oct 1;15:34273. doi: 10.1038/s41598-025-16523-2 (PMC12489129; doi:10.1038/s41598-025-16523-2)
Supplement: Supplementary file 8 — Supplementary Material 8 [file 41598_2025_16523_MOESM8_ESM.pdf]

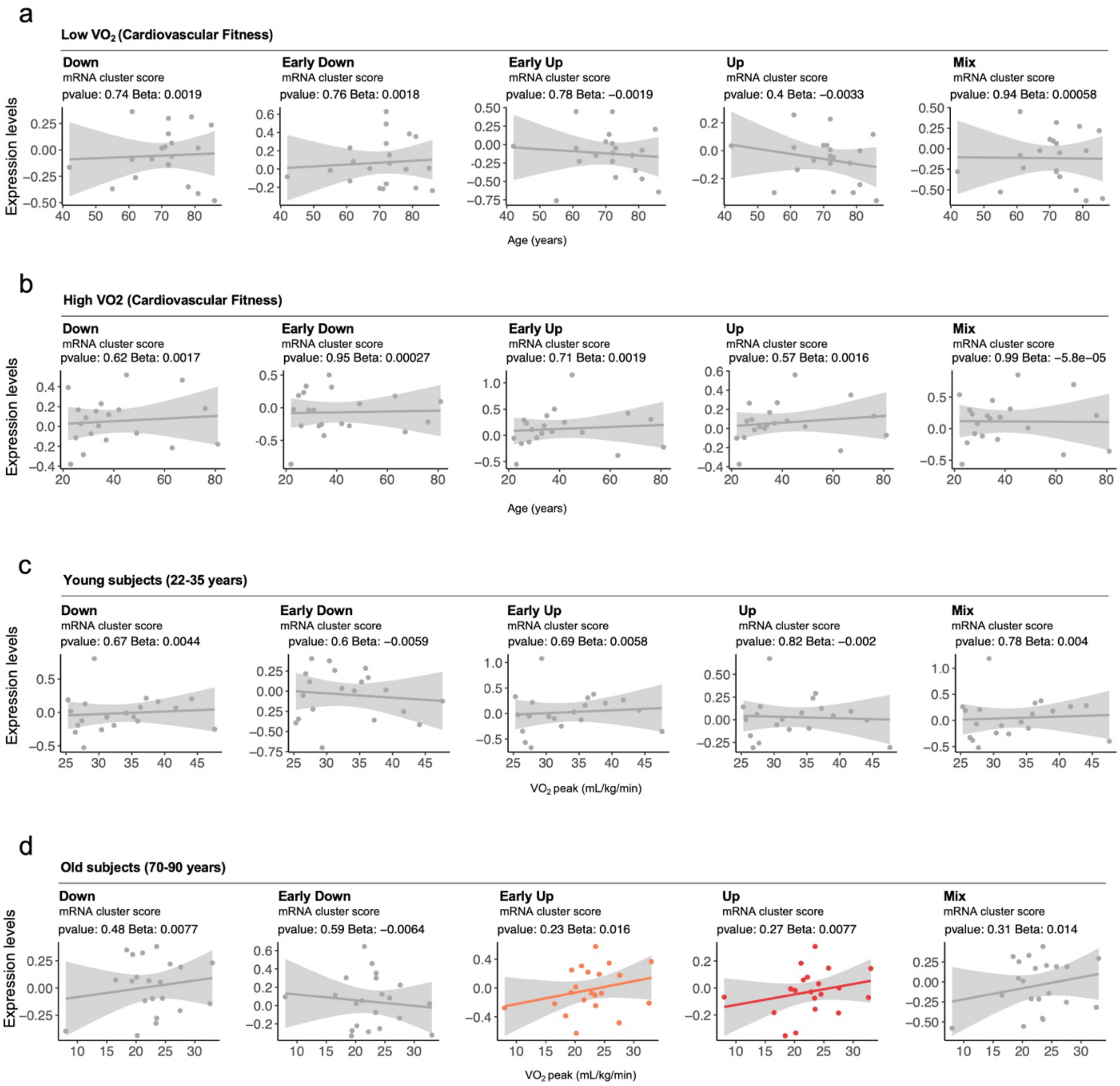

Supplementary Material 8. mRNA cluster validation using Data in healthy human aging. Data were collected from GESTALT - the Genetic and Epigenetic Signatures of Translational Aging (human muscle biopsies) by a previous study (Donega et al., 2024). a) Validation of myogenesis clusters in aging subjects with low VO_2 (cardiovascular fitness). b) Validation of myogenesis clusters in aging subjects with high VO_2 (cardiovascular fitness). c) Validation of myogenesis clusters based on fitness (VO_2 levels) in young subjects aged 22–35 years. d) Validation of myogenesis clusters based on fitness (VO2 levels) in older subjects aged 70+

years. The colored clusters scores "Early-Up" and "Up" represent the most significant results, although they do not reach statistical significance (p=0.23 and p=0.27, respectively).

Donega, S., Banskota, N., Gupta, E., Gonzalez-Freire, M., Moore, A. Z., Ubaida-Mohien, C., Munk, R., Zukley, L., Piao, Y., Bergeron, C., Bergeron, J., Bektas, A., Zampino, M., Stagg, C., Indig, F., Hartnell, L. M., Kaileh, M., Fishbein, K., Spencer, R. G., . . . Ferrucci, L. (2024). Skeletal Muscle mRNA Splicing Variants Association With Four Different Fitness and Energetic Measures in the GESTALT Study. *J Cachexia Sarcopenia Muscle*. https://doi.org/10.1002/jcsm.13603
