# Supplementary material for: In-vitro human myogenesis model reveals novel mRNA alternative splicing isoforms
Source: Sci Rep. 2025 Oct 1;15:34273. doi: 10.1038/s41598-025-16523-2 (PMC12489129; doi:10.1038/s41598-025-16523-2)
Supplement: Supplementary file 9 — Supplementary Material 9 [file 41598_2025_16523_MOESM9_ESM.pdf]

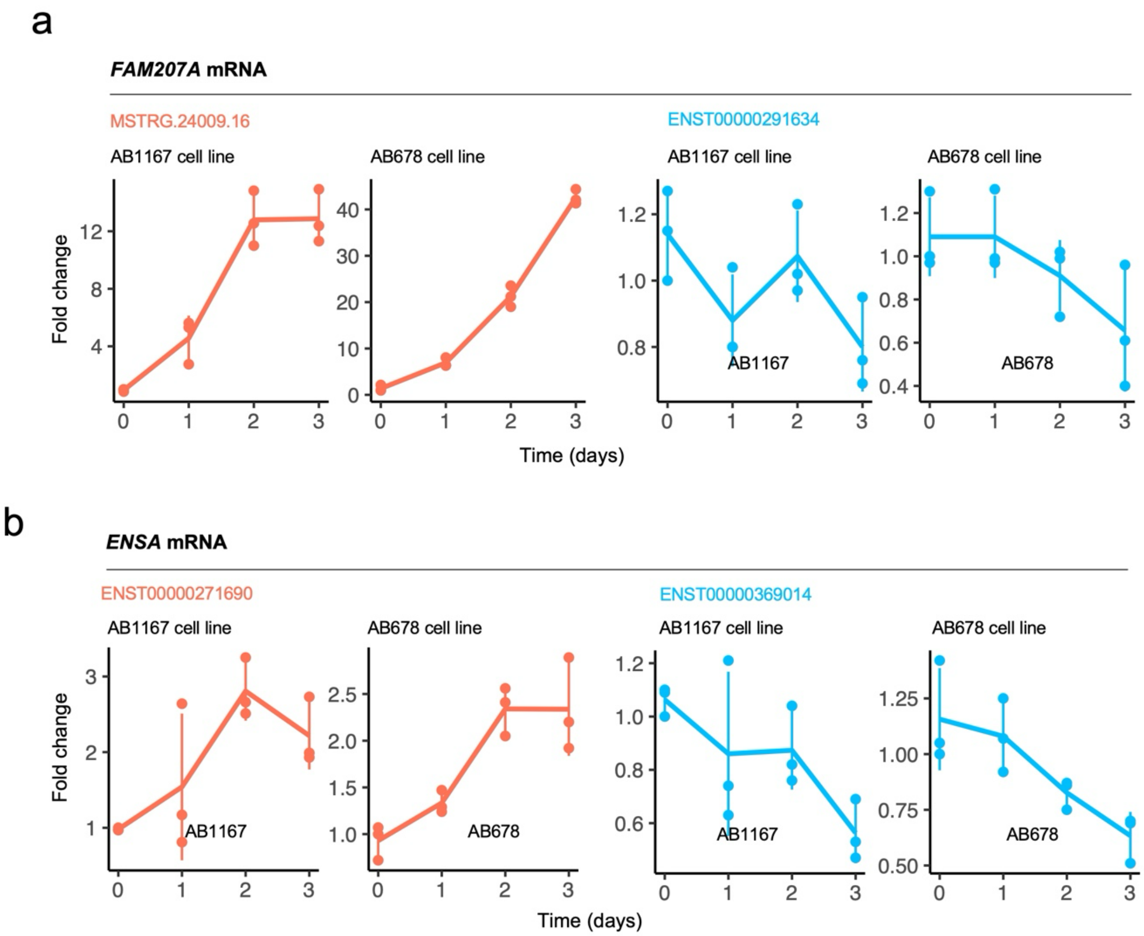

Supplementary Material 9. RT-qPCR validation for DTU in AB1167 and AB678 cells. Two isoforms are validated, normalized to *GAPDH* mRNA levels: a) *FAM207A* mRNA and b) *ENSA* mRNA
