# Supplementary material for: In-vitro human myogenesis model reveals novel mRNA alternative splicing isoforms
Source: Sci Rep. 2025 Oct 1;15:34273. doi: 10.1038/s41598-025-16523-2 (PMC12489129; doi:10.1038/s41598-025-16523-2)
Supplement: Supplementary file 10 — Supplementary Material 10 [file 41598_2025_16523_MOESM10_ESM.pdf]

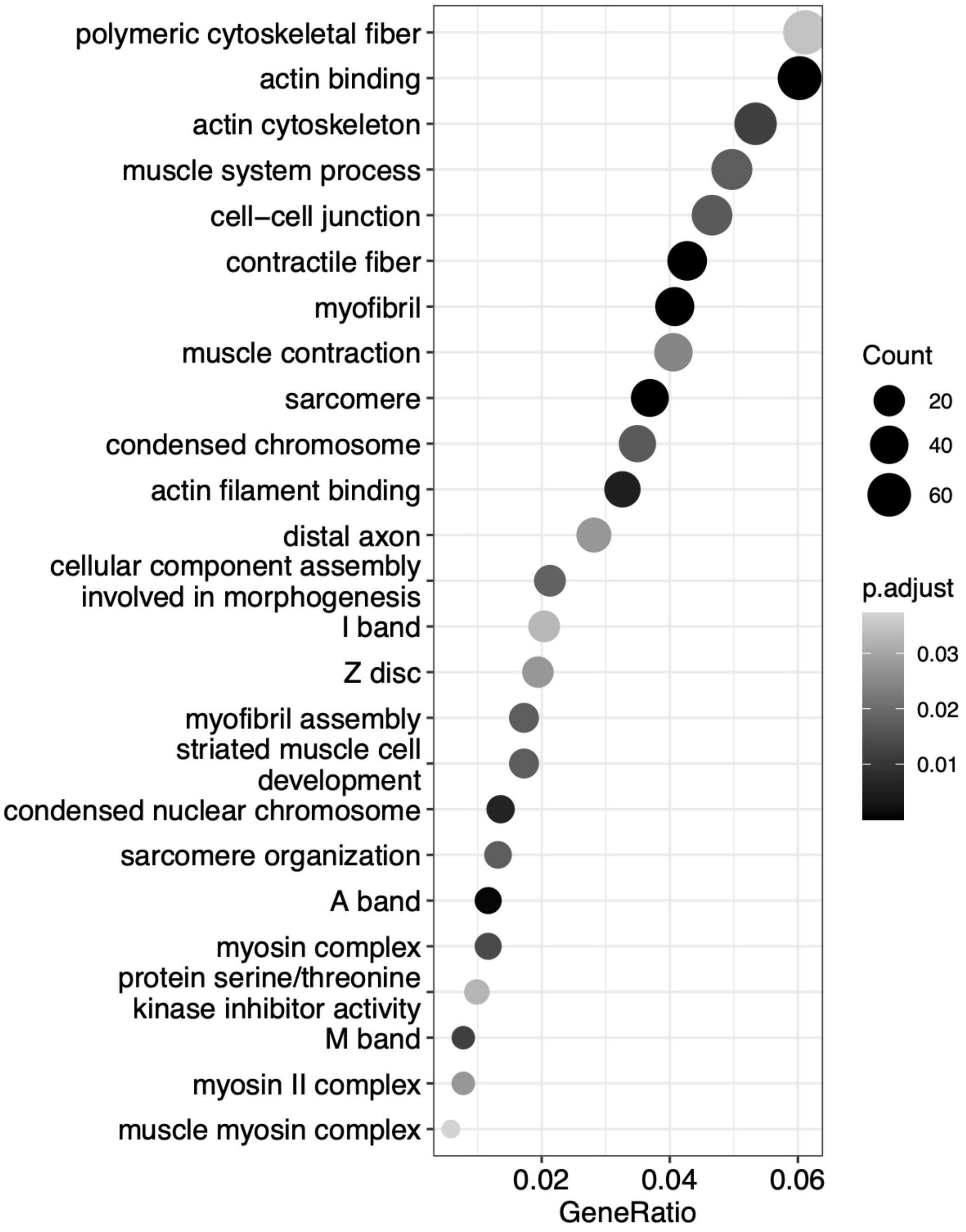

Supplementary Material 10. Pathway analysis for the DTU analysis using Over-Representation Analysis (ORA). All pathways associated to myogenesis are visualized
