# Supplementary material for: In-vitro human myogenesis model reveals novel mRNA alternative splicing isoforms
Source: Sci Rep. 2025 Oct 1;15:34273. doi: 10.1038/s41598-025-16523-2 (PMC12489129; doi:10.1038/s41598-025-16523-2)
Supplement: Supplementary file 11 — Supplementary Material 11 [file 41598_2025_16523_MOESM11_ESM.pdf]

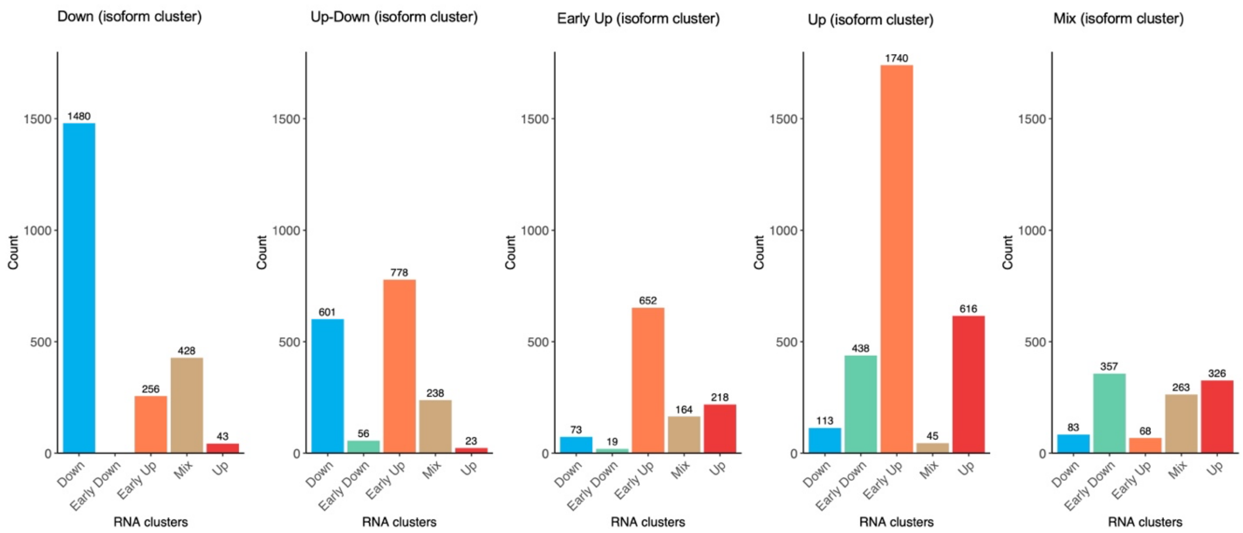

Supplementary Material 11. Distribution of RNAs across splicing isoform clusters relative to mRNA clusters. Five isoform clusters are displayed in a single row, arranged in the same order as in main Figure 7. Each plot illustrates the number of RNAs from each RNA cluster within the respective isoform cluster, confirming a strong correspondence between isoform and RNA clusters
